# Tissue‐Resident Memory T Cells: Pioneering New Avenues in Cancer Immunotherapy

**DOI:** 10.1002/cam4.71429

**Published:** 2025-12-08

**Authors:** Yao Xiao, Qi‐Chao Yang, Zhi‐Jun Sun

**Affiliations:** ^1^ State Key Laboratory of Oral & Maxillofacial Reconstruction and Regeneration, Key Laboratory of Oral Biomedicine Ministry of Education, Hubei Key Laboratory of Stomatology, School & Hospital of Stomatology, Frontier Science Center for Immunology and Metabolism, Taikang Center for Life and Medical Sciences Wuhan University Wuhan China; ^2^ Department of Oral Maxillofacial‐Head Neck Oncology, School & Hospital of Stomatology Wuhan University Wuhan China

**Keywords:** cancer, immunotherapy, phenotype markers, tissue‐resident memory T cell, transcription factors

## Abstract

**Background:**

Tissue‐resident memory T cells (T_RM_) are a specialized subset of memory T cells that reside in nonlymphoid tissues and draining lymph nodes, playing a critical role in immune surveillance and tumor immunity. Tumor‐infiltrated T_RM_ have emerged as key players in immune checkpoint blockade (ICB) therapy, contributing to early therapeutic responses and tumor control.

**Aims:**

This review aims to comprehensively summarize the identification, functional roles, and regulatory mechanisms of T_RM_ in the context of tumor immunity, and to explore innovative strategies for enhancing T_RM_‐mediated anti‐tumor responses.

**Methods:**

We reviewed recent literature on T_RM_ biology, focusing on their transcriptional regulation, phenotypic characteristics, and interactions within the tumor microenvironment. We also reviewed current strategies for modulating T_RM_ function to improve cancer immunotherapy outcomes.

**Results:**

T_RM_ can remodel the tumor microenvironment, suppress tumor progression, and enhance ICB efficacy. Their function is tightly regulated by transcription factors and phenotypic molecules. Targeted manipulation of these regulatory mechanisms has shown potential in enhancing T_RM_/T_RM_‐like cell activity and overcoming resistance to ICB therapy.

**Conclusion:**

T_RM_ represent a promising target for cancer immunotherapy due to their potent anti‐tumor functions and responsiveness to ICB. Understanding their biology and regulatory networks opens new avenues for therapeutic intervention.

**Abbreviations**
AhR: aryl hydrocarbon receptor
Bhlhe40: basic helix–loop–helix family member e40
Blimp‐1: B lymphocyte maturation protein‐1
Eomes: eomesodermin
FABP4/FABP5: fatty‐acid‐binding proteins 4/fatty‐acid‐binding proteins 5
GZMB: granzyme B
Hobit: homolog of Blimp‐1 in T cells
Id2/Id3: inhibitor of DNA binding 2/inhibitor of DNA binding 3
IFN‐γ: interferon‐γ
IL‐15: interleukin 15
IL‐2: interleukin 2
IL‐7: interleukin 7
KLF2: KLF transcription factor 2
KLRG1: killer cell lectin‐like receptor G1
MDSC: myeloid‐derived suppressor cell
Nr4a1: nuclear receptor subfamily 4 group A member 1
PAK4: p21‐activated kinase 4
PD‐1: programmed cell death protein 1
PD‐L1: programmed cell death protein ligand 1
PPAR: peroxisome proliferator‐activated receptor
PRF: perforin
Runx3: RUNX family transcription factor 3
S1PR1: sphingosine‐1‐phosphate receptor 1
T‐bet: T‐box transcription factor 21
TCF‐1: T cell factor 1
T_CM_
: central memory T cell
T_EM_
: effector memory T cell
TGF‐β: transforming growth factor‐β
TIL: tumor‐infiltrating lymphocyte
TME: tumor microenvironment
Treg: regulatory T cell
T_RM_
: tissue‐resident memory T cell

## 1 Introduction

CD8^+^tissue‐resident memory T cells (T_RM_) are an important part of the immune system, acting as guardians of peripheral tissues and draining lymph nodes [1]. Upon pathogen invasion, T_RM_ are generated to provide local resistance; in many tissues they can persist for extended periods, although their longevity is context‐dependent and varies by anatomical site [2, 3, 4, 5, 6]. With the progress of research in recent years, T_RM_ have been found to exist in many types of tumors and have a powerful capacity to fight against cancer [7, 8, 9]. Furthermore, these cells have been found to have a substantial positive impact on the prognosis of patients and act as responders in immune checkpoint blockade (ICB) therapy [7, 9, 10]. These breakthroughs have inspired a new line of inquiry into how to address resistance to ICB therapy, a growing concern in the field of tumor immune research. By virtue of their unique immunological properties, T_RM_ offer an invaluable opportunity to tackle this challenge and pave the way for more effective cancer treatments.

T_RM_ effector function is dynamically regulated by a suite of molecules that represent promising therapeutic targets [9]. Transcription factors like Runx3 govern residency and cytotoxic programming, while surface markers such as CD103 and CD69 mediate tissue retention and effector function [9, 11]. Furthermore, cytokines including TGF‐β and IL‐15 critically support survival and functional adaptation [12, 13]. Deliberate manipulation of these mechanisms, for instance by promoting residency programs or countering inhibitory signals, enables the design of novel strategies to amplify T_RM_ activity [14].

This review summarizes recent progress in T_RM_ research. It covers the basics of T_RM_ identification and generation, discusses their functions and regulatory mechanisms, and focuses on their potential in cancer therapy. The aim is to aid in understanding T_RM_ biology and inform the development of new cancer immunotherapies. This review focuses exclusively on CD8^+^ T_RM_; CD4^+^ T_RM_ are mentioned only when the cited study explicitly examined that subset.

## 2 Fundamental Characteristics of T_RM_

### 2.1 Definition and Phenotypic Features

T_RM_ are defined by their tissue residency without recirculation [14]. This property is typically assessed through migration assays, including: Parabiosis surgery [15, 16], This technique involves surgically joining two congenic mice, enabling them to share a common bloodstream; circulating T cells migrate between the two mice, whereas T_RM_ remain tissue‐resident and rarely recirculate. Tissue transplantation [4, 17], grafting tissues containing traceable lymphocytes are transplanted, circulating T cells from the donor tissue recirculate into the graft, while T_RM_ remain localized, allowing for their distinction. Administration of the S1P1 functional antagonist FTY720 blocks lymphocyte egress from lymphoid organs and reduces circulating T‐cell numbers; persistence of a T‐cell population in the tissue under these conditions indicates residence independent of recirculation [17, 18]. Moreover, in organ transplantation experiments, the donor organ contains not only donor‐derived T_RM_ but also recipient‐derived T cells that are progressively acquiring T_RM_ phenotypes [4]. To detect donor‐derived T_RM_, FTY720 can be used to inhibit T cell egress from lymphoid organs, thereby reducing the infiltration of new recipient T cells into the graft, which may facilitate the identification of persisting donor‐derived T_RM_ [4]. However, FTY720 mainly inhibits S1P‐dependent egress from lymph nodes and has no effect on S1P‐independent pathways [18].

When migration assays are not feasible, intravascular antibody labeling/depleting and phenotype marker detection can be used instead [19]. Intravascular antibody labeling/depleting [20, 21]: Antibodies targeting circulating T cells are injected intravascularly. These antibodies label and deplete circulating T cells but do not affect T_RM_, which are shielded from intravascular exposure. Intravascular labeling cannot reliably separate circulating T cells from those that reside in tissues unless parabiosis or tissue transplantation is also performed [14]. A complementary approach, exemplified by low‐dose subcutaneous alemtuzumab, is to titrate the antibody so that it eliminates circulating T cells yet leaves sessile tissue‐resident T cells largely untouched [21].

Phenotype marker detection [2, 11, 22]: Surface expression of CD103 and CD69 is commonly used to identify T_RM_ cells, as these molecules mediate tissue retention; CD49a can provide additional supporting evidence in certain organs, nuclear transcription factors such as Runx3, Blimp‐1 and Hobit control T_RM_ differentiation and/or function, used as supplementary indicators. However, there is no single marker that could define *bona fide* T_RM_ cells [14]. CD103 is not expressed on some T_RM_, liver T_RM_ are typically CD103‐negative [23]. CD69 is also expressed on activated T cells [24], furthermore, it promotes the generation of T_RM_ in a tissue‐dependent manner: it is essential in the kidney, beneficial in the lung, but redundant in the intestine [25]. CD49a is also expressed on some circulating T cells [26]. The transcription factors Runx3, Blimp‐1, and Hobit are also differentially expressed in T_RM_ from different tissues [11]. Therefore, expression of these phenotype markers may only suggest that cells are T_RM_‐like (Table 1).

**TABLE 1:** Phenotype markers of T_RM_.

Marker		Function	References
Retention	CD103	CD103 bind to E‐Cadherin facilitate T_RM_ retention in tissue and CD103 mediate cytotoxicity of T_RM_	[27]
	CD49a	CD49a facilitate T_RM_ retention in tissue; CD49a expression marks CD8^+^ T_RM_ poised for IFN‐γ production in human skin	[28, 29, 30]
	CD69	CD69 inhibits surface expression of the S1P receptor which regulating T cell tissue egress	[31, 32]
	CD44	CD44 bind to hyaluronic acid and another matrix component	[1]
Recruitment	CXCR6	Promote T_RM_/ T_RM_‐like cells localization in ovarian cancer and skin through CXCR6/CXCL16 axis	[33, 34]
	CXCR8	CXCR8 plays a crucial role in mucosal vaginal immunity by promoting the mobilization of functional protective CD8^+^T_EM_ and CD8^+^T_RM_ against herpes infection through CXCR8/CXCR17 pathway	[35]
	CXCR3	CXCR3 is critical for T cell migration to uninfected salivary glands but not affect T cell migration to MCMV infected salivary glands; CXCR3 knockout reduced CD103^+^CD69^+^T cell in skin	[34, 36]
Metabolism	FABP4/FABP5	FABP4 and FABP5 facilitate T_RM_ survival and function by enabling the uptake of exogenous FFAs and supporting mitochondrial oxidative metabolism	[37, 38]
Immunosuppression	PD‐1/CTLA4/TIM3	Mediate T_RM_ immune tolerance	[7]

**FIGURE 1:**
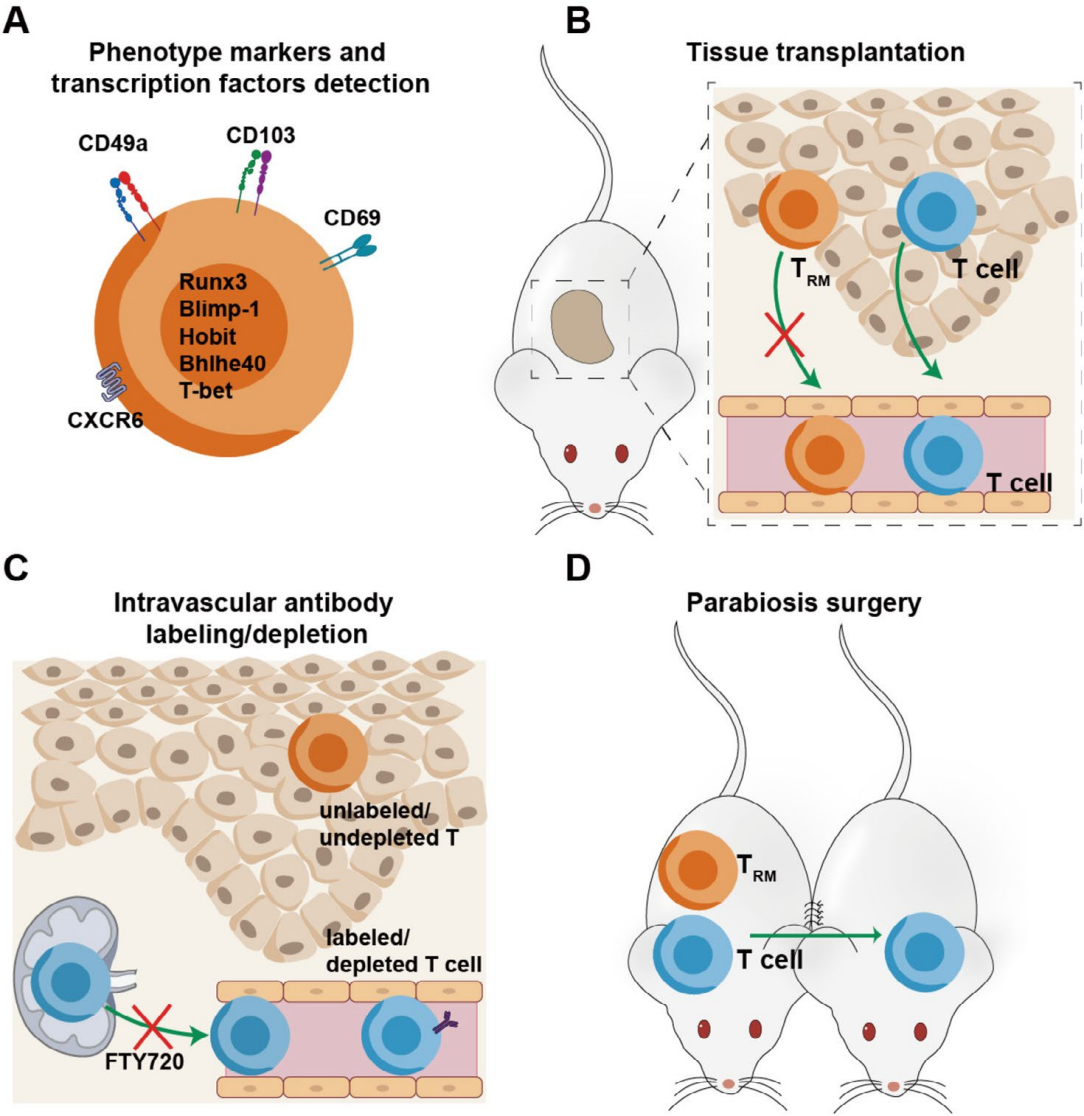
The approaches for identifying the tissue‐resident memory feature of T cells. (A) Phenotype markers and transcription factors detection, applied flow cytometry and/or RNA‐seq to detect T_RM_ markers. None of the listed markers is uniquely expressed by T_RM_, and individual T_RM_ cells may display only a subset of these molecules depending on tissue context. (B) Transplantation of tissues with trackable lymphocytes, T_RM_ will not recirculate to the graft. (C) Intravascularly injected T cell labeled/depleted antibody and FTY720 administration; circulating T cells will be labeled/depleted, but T_RM_ could not be labeled/depleted. FTY720 blocks S1P‐dependent lymphocyte egress from lymphoid organs, rapidly reducing circulating T cells. (D) Conjoin the skin of two congenic mice; T_RM_ will not migrate from one to another.

Moreover, in the tumor microenvironment, a proportion of CD69^+^CD103^+^ T cells may reflect exhausted T cells rather than genuine T_RM_, making it difficult to distinguish T_RM_ identity based solely on surface markers [14]. Gavil et al. [39] showed that TIM3 and CD39 better reflect T cell residency within tumors than classical T_RM_ markers. In addition, multiple studies have demonstrated the tumor‐reactive role of CD39^+^ T_RM_ [40, 41, 42, 43]. Thus, in scenarios where migration assays cannot be performed and only phenotype marker detection is available, more precise markers should be developed, and multiple markers should be used to identify T_RM_ cells (Figure 1).

### 2.2 Differentiation and Maintenance Mechanisms of T_RM_

T_RM_ generation is controlled by several cytokines: TGF‐β drives CD103 and CD69 expression [2, 13], the key sources of TGF‐β include CD103^+^ DCs, keratinocytes, and fibroblasts [2, 13, 44, 45]. IL‐15 supports T_RM_ survival and effector function [13], Membrane‐bound IL‐15‐IL‐15Rα complexes are trans‐presented by Batf3‐dependent CD103^+^/CD8α^+^ cDCs to CD8^+^ T cells during priming [46]. In tissues such as skin, keratinocytes produce IL‐15 to support T_RM_ survival and retention [47]. IL‐12 induces CD49a expression on CD8^+^ T cells, which promotes their persistence in tissue and interaction with the extracellular matrix [48]. IL‐12 can be produced by CD8α^+^ and CD103^+^ conventional dendritic cells (DCs) in draining lymph nodes during initial T cell priming [46].

**FIGURE 2:**
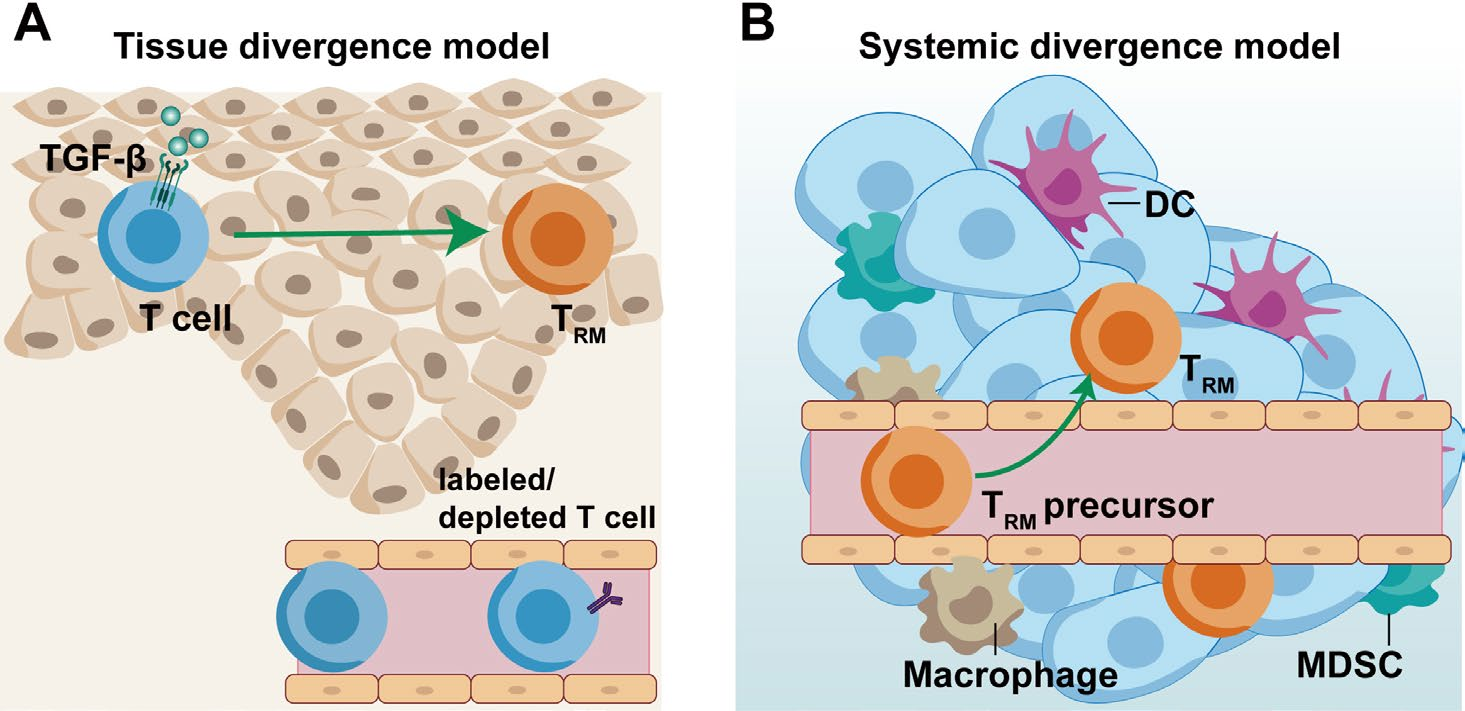
The generation model of T_RM_. (A) Tissue divergence model, only when T cells enter peripheral tissues do they acquire the potential to differentiate into T_RM_. (B) Systemic divergence model, T cells acquire a part of T_RM_ phenotype and acquire the potential to differentiate into T_RM_ before entering peripheral tissues.

Two developmental models have been proposed in T_RM_ differentiation [49] (Figure 2). In the tissue divergence model, T cells enter the periphery first and then acquire T_RM_ potential [6]. In the systemic divergence model, T cells acquire T_RM_ potential within secondary lymphoid organs before egress, allowing them to arrive in tissues and tumors already programmed for residence [50]. Tumor‐infiltrating T_RM_ may follow the systemic divergence model. The expression of CD103 on CD8^+^T cells enhances their penetration into tumors, where they exert potent anti‐tumor effects [51].

## 3 Functional Roles and Phenotypic Determinants of T_RM_ in Tumor Immunity

### 3.1 T_RM_ ‐Mediated Tumor Control

T_RM_ cells express low levels of perforin and granzymes under steady‐state conditions, thereby limiting tissue damage [11, 12]. Upon antigen re‐encounter or exposure to inflammatory cues such as IL‐15, these cells rapidly up‐regulate cytotoxic molecules and acquire potent killing capacity [11, 12]. Throughout the long‐term process of tumor development, the immune system, particularly T_RM_, plays a critical role in surveilling and controlling malignant cells [52, 53]. This dynamic interplay, which may persist for years, involves the rapid elimination of emerging tumors and the durable suppression of residual disease [52, 54, 55, 56, 57].

At the primary site, pre‐installed T_RM_ cells can abort tumor outgrowth shortly after vaccination. Galvez‐Cancino et al. [54] in mice showed that a single intradermal vaccination rapidly generates skin T_RM_ cells which significantly inhibit local outgrowth of B16F10 melanoma. This protection is independent of circulating CD8^+^ T cells and provides the first direct evidence that “pre‐installed” T_RM_ cells can block tumor initiation at the primary site [54]. In a parallel study, Enamorado et al. [55] using mouse models demonstrated that vaccine‐induced T_RM_ cells can mediate anti‐tumor immunity independently of circulating CD8^+^ T cells. Mice were infected with rVACV‐OVA via skin scarification; thirty days later, when T_RM_ cells were fully established in skin and lung, they were challenged intradermally with B16‐OVA melanoma. FTY720 was administered to block T cell egress from lymph nodes, ensuring that only T_RM_ cells could contribute to protection [55]. And T_RM_ cells alone were found to significantly delay tumor outgrowth [55].

The same T_RM_ pool then switches to long‐term, sub‐clinical surveillance [52]. Park et al. [52] extended the murine observation window and found that the same T_RM_ pool did not wane. Instead, it remained permanently anchored in the epidermis, performing “multi‐year” surveillance of microscopic disease. Intermittent tumor DNA could still be detected, yet T_RM_ cells kept lesions sub‐clinical by repeated antigen encounter and sustained IFN‐γ/TNF‐α secretion, turning an early blockade into durable control [52]. Thus, T_RM_ provides both rapid elimination of emerging malignant cells and durable immune surveillance that keeps residual disease sub‐clinical.

Even after surgery, experimentally induced vitiligo can expand T_RM_ and avert local relapse. Malik et al. [56] used a murine postsurgical model to show that following primary‐tumor excision and antibody‐mediated depletion of regulatory T cells, the resulting experimentally induced vitiligo drives robust expansion of CD103^+^ skin T_RM_. Detached from lymphatic recirculation, these cells form high‐density “immune sentinels” in depigmented hair follicles after surgery and almost completely prevent recurrence upon contralateral rechallenge, proving that therapeutically induced T_RM_ can also provide long‐lived local immunity [56].

Extending beyond skin, T_RM_ cells in draining lymph nodes intercept metastatic tumor cells. Molodtsov et al. [57] demonstrate that T_RM_ established within murine regional lymph nodes (RLNs) act as a frontline barrier against melanoma metastasis. After primary tumor excision and vitiligo induction, tumor‐specific CD69^+^CD103^+^ T_RM_ persist for months in RLNs; parabiosis and rechallenge experiments show these cells neither recirculate nor rely on circulating memory T cells, yet their presence alone prevents metastatic seeding in the LN [57]. scRNA‐seq further identifies a LN‐T_RM_ signature (high IL‐7R, CXCR6, IFN‐γ) that independently predicts better survival in metastatic melanoma, underscoring the critical role of LN‐resident memory in suppressing lymph‐node metastatic outgrowth [57].

### 3.2 T_RM_ Phenotype Markers in T Cell‐Mediated Tumor Control

The phenotypic molecules on T_RM_ surface are far more than identity badges; they are functionally essential [58]. They orchestrate T‐cell recruitment to the tumor, anchor the cells within the microenvironment, and heighten antigen sensitivity, together forming the core mechanism by which T_RM_/T_RM_‐like cells execute antitumor immunity [27, 28, 33].

T_RM_ characteristically express elevated levels of CXCR6, enabling them to respond to the chemokine CXCL16, a feature that distinguishes them from circulating memory T cells [59, 60]. In the tumor microenvironment, CD103^+^CD8^+^ T_RM_‐like cells also utilize the CXCL16–CXCR6 axis to accumulate at tumor sites [33].

Adhesion molecules on the surface of T_RM_, such as CD103, are associated with T cell anchoring within the tumor microenvironment and with infiltration into tumor epithelial islets [61, 62]. CD103–E‐cadherin binding facilitates CD103^+^CD8^+^T cell infiltration, and CD103^+^CD8^+^T cells have been proven to accumulate in intra‐tumor islets, associated with a favorable prognosis in ovarian cancer [63], non‐small cell lung carcinoma [61], and oral cancer [64]. Anti‐CD103 treatment reduces CD8^+^T cell infiltration [62]. CD49a also serves as an adhesion molecule and is associated with infiltration; anti‐CD49a treatment results in the loss of CD8^+^T cell infiltration [29, 65].

Moreover, both CD103 and CD49a have been proven to be associated with the cytotoxicity of T_RM_‐like cells. CD103 interaction with E‐cadherin promotes lytic granule polarization [66], and CD103^+^CD8^+^ T cells express higher levels of cytotoxic molecules such as granzyme B and perforin than their CD103^−^CD8^+^T cells counterparts [7, 67]. The expression of these cytotoxic molecules in CD103^+^CD8^+^T cells can be impaired after anti‐CD103 treatment [61, 66]. CD49a marks a functionally specialized subset of T_RM_ cells with potent cytotoxic potential. CD49a^+^CD103^+^CD8^+^ T_RM_‐like cells exhibit higher expression of perforin and granzyme B, produce IFN‐γ, and respond robustly to IL‐15 stimulation, distinguishing them from their CD49a^−^CD103^+^CD8^+^ T_RM_‐like cell counterparts [28, 48]. Genetic deletion of CD49a (*Itga1*) in mouse models leads to reduced persistence of T_RM_ cells and impairs their effector function, including decreased IFN‐γ production and diminished viral clearance upon antigen challenge [48].

Additionally, adhesion molecules increase the sensitivity of TCR to tumor antigens. Cytotoxic T cells use the TCR‐MHC I interaction to recognize tumor antigens. Interaction at the immune synapse is facilitated by the LFA‐1‐ICAM‐1 complex, allowing T cells to adhere to specific tumor cells [68]. Recent research has revealed that CD103‐E‐cadherin interactions enhance TCR recognition, making the cells more sensitive to detecting tumor antigens [27].

### 3.3 Immune Recruitment and Collaboration

Researchers have been astounded by the impressive anti‐tumor effect of T_RM_, which goes beyond individual cells [9]. T_RM_ cells play a sentinel role in tumor immunity by responding rapidly upon antigen‐specific activation and initiating a localized immune cascade [69]. Upon antigen recognition, T_RM_ cells secrete large amounts of IFN‐γ and TNF‐α, which act locally to activate dermal DCs and promote their maturation and migration to draining lymph nodes [69]. These activated DCs, in turn, cross‐present tumor‐derived antigens to naïve or circulating CD8^+^ T cells, leading to the expansion of new cytotoxic T cell clones targeting both the original and neoantigens expressed by the tumor [69].

In addition to DC activation, T_RM_ reactivation also triggers innate immune responses [70]. Schenkel et al. [70] demonstrated that T_RM_‐derived IL‐2 upregulates granzyme B expression in natural killer (NK) cells and bystander memory CD8^+^ T cells, enhancing their cytolytic potential. This IL‐2‐dependent signaling is essential, as blockade of the IL‐2 receptor abrogates the activation of these effector cells [70].

CD103^+^CD8^+^T_RM_‐like cells are closely associated with tertiary lymphoid structures (TLS) in multiple tumor types [71]. Traditionally, CD4^+^T follicular helper (TFH) cells, particularly CXCL13+ TFH cells, have been implicated in TLS formation through B cell recruitment [71]. However, recent studies have revealed that CD103^+^CD8^+^T cells with T_RM_‐like features can also recruit B cells and facilitate TLS formation via CXCL13 production [71]. Compared to CD103^−^CD8^+^T cells, CD103^+^CD8^+^T cells express significantly higher levels of CXCL13 [71]. CXCL13 secreted by these T_RM_‐like CD8^+^T cells promotes B cell recruitment and TLS formation in the tumor microenvironment [71]. The coexistence of TLS and CXCL13^+^CD103^+^CD8^+^ T_RM_‐like cells in tumor tissues is associated with improved response to anti‐PD‐1 therapy [71, 72]. Thus, these T_RM_‐like CD8^+^T cells may serve as immune sentinels that contribute to local and systemic anti‐tumor immunity, particularly in the context of ICB therapy.

### 3.4 T_RM_ ‐Like Cells in Immune Checkpoint Blockade Therapy

T_RM_‐like cells are associated with the efficacy of ICB therapy [10]. In various types of tumors, T_RM_‐like cells are enriched in patients who respond to ICB therapy, such as breast cancer [73], melanoma [74], lung cancer [75] as well as head and neck cancer [76]. Emerging work reveals that T_RM_‐like cells are not static during ICB but can undergo functional reprogramming and clonal expansion [7]. For example, Luoma et al. [10] reported that in a neoadjuvant ICB trial, ITGAE^+^ (CD103^+^) T_RM_‐like CD8^+^ T cells expanded markedly within the tumor during the short course of treatment. These cells expressed high levels of cytotoxicity‐related genes (e.g., *GZMB*, *PRF1*) and effector cytokines (e.g., *IFNG*, *TNF*), indicating potent effector capacity [10]. Rather than displaying canonical exhaustion signatures, the T_RM_‐like population remained highly activated and proliferative after ICB, suggesting they can be “re‐awakened” to participate in antitumor immunity [10].

TCR sequencing combined with single‐cell transcriptomics further showed that the T_RM_‐like clones expanded during ICB can persist for years in tumor, blood, and even skin, implying long‐term immune surveillance [77].

### 3.5 T_RM_ Correlated With Poor Prognosis in Some Certain Types of Tumors

**FIGURE 3:**
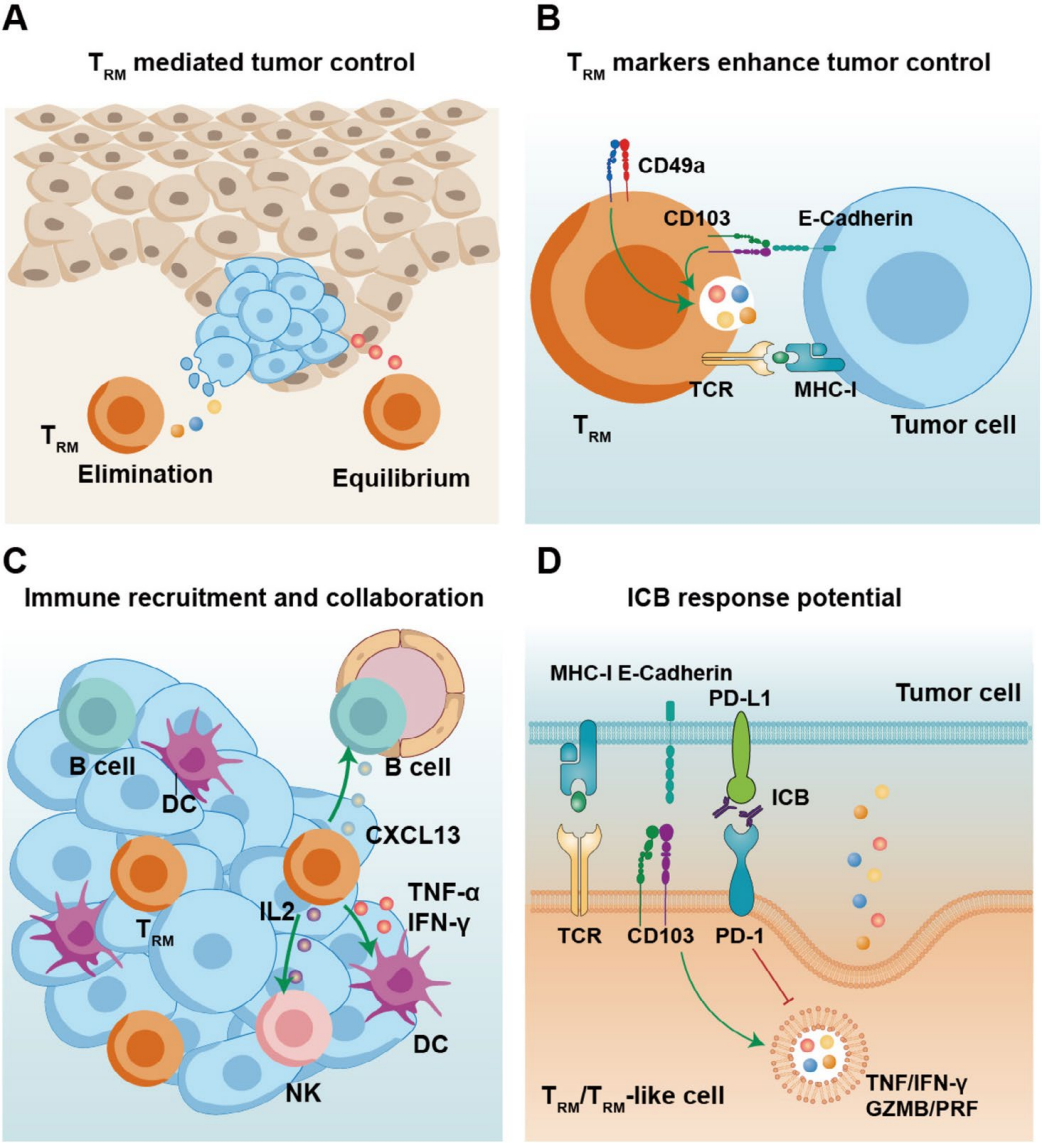
Functional roles and phenotypic determinants of T_RM_ in tumor immunity. (A) Local immune surveillance by T_RM_. (B) T_RM_ phenotype markers in T cell‐mediated tumor control. (C) T_RM_ expand the immune response by working with other immune cells to combat tumor cells through secretory cytokines. (D) T_RM_/T_RM_‐like cells in ICB therapy.

In human cutaneous squamous cell carcinoma (cSCC) [78], clear cell renal cell carcinoma (ccRCC) [79], glioblastoma, and colorectal cancer [80], high infiltration of T_RM_ or T_RM_‐like CD103^+^ T cells is associated with shorter survival. Lai et al. [78] validated this link in human cSCC: CD8^+^CD69^+^CD103^+^ T cells isolated from primary tumors produced significantly more IL‐10 and expressed higher levels of CD39, CTLA‐4, and PD‐1 than their CD103^−^ counterparts, suggesting a functionally suppressive phenotype. In human ccRCC, Sanders et al. [79] found that CD103^+^ lymphocytes are enriched in lung metastases and that high intratumoral CD103^+^ density correlates with poorly differentiated grade, distant metastasis, and shorter overall survival in two independent patient cohorts plus TCGA‐KIRC data. Gabriely et al. [80] provided mechanistic evidence in murine melanoma: CD103^+^CD8^+^T cells from tumor‐bearing mice expressed IL‐10, CD25, and CTLA‐4, suppressed CD8^+^T‐cell proliferation in vitro in a partly PD‐1/PD‐L1–dependent manner, and accelerated tumor growth upon adoptive transfer in vivo. Concurrent analysis of TCGA datasets further associated high *ITGAE* (CD103) mRNA expression with poor survival in glioblastoma and colorectal cancer, although functional studies specifically addressing CD103^+^ T_RM_ in colorectal cancer are still lacking [80]. Collectively, these studies establish that T_RM_ can adopt a regulatory, pro‐tumorigenic phenotype and correlate with adverse clinical outcomes in certain tumor types. These findings may be related to the heterogeneity of T_RM_. Han et al. [77] identified that an *IFNG*‐expressing T_RM_‐like subset independently predicted longer overall survival, while a *TOX*‐expressing T_RM_ subcluster was associated with poorer outcome, indicating that T_RM_‐like subpopulations differ functionally and prognostically. Therefore, when designing T_RM_‐based therapeutic strategies or using T_RM_ as prognostic markers, their functional heterogeneity must be taken into account (Figure 3).

**TABLE 2:** Transcription factors of T_RM_.

Transcription factors		Function	References
Activation	Runx3	Programed T_RM_ tissue residency by increase CD103 and CD69 expression; Drive T cell effector function by increase perforin, granzyme B expression	[81, 82]
	Blimp‐1	Suppresses tissue egress/homing genes (*S1pr1*, *Ccr7*); induces granzyme B	[83, 84, 85]
	Hobit	Suppresses tissue egress genes (e.g., *S1pr1*, *Klf2*)	[83, 84, 86]
	AhR	Activated the effector associated gene *Gzmb*, *Itgae* and *Prdm1* expression while suppress the gene mediates T cells tissue egress and lymph tissue homing such as *Klf2* and *S1pr5*	[87, 91]
Metabolism	Bhlhe40	Bhlhe40 maintains function and survival of T_RM_; Programs T_RM_ and TIL mitochondrial metabolism. Promote CD103 and CD69 expression	[92]
Maintenance	Eomes	Eomes limits T_RM_ formation but supports the maintenance of established T_RM_, in part by inducing Bcl‐2 expression	[3, 86]
	T‐bet	T‐bet were required for S1pr5 induction impede T_RM_ formation, but T‐bet expression is required for IL‐15‐mediated CD8^+^CD103^+^ T_RM_ survival	[13, 93]
Homeostasis	Id2/Id3	Maintain T_RM_ homeostasis & memory phenotype	[88]
	Nr4a1	Nr4a1 promotes tissue residence of murine CD8^+^T_RM_ but contributes to T cell dysfunction by suppressing effector function	[94, 95]

## 4 The Transcriptional Regulation of T_RM_

**FIGURE 4:**
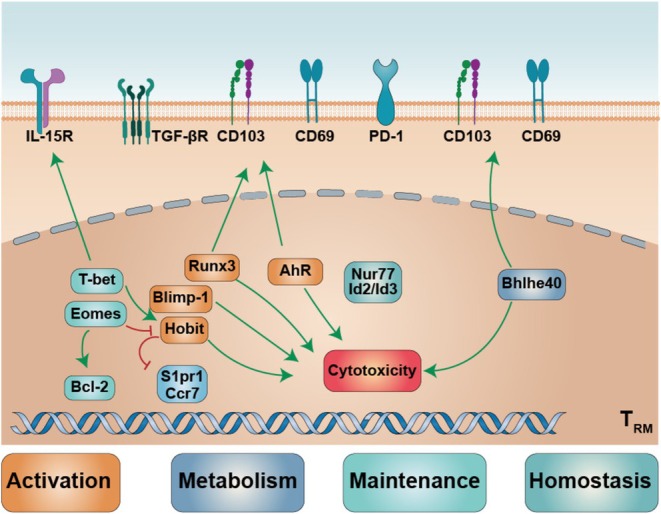
The transcription factors and their effect on T_RM_ function. The transcription factors for activation, metabolism, maintenance, and homeostasis.

Several transcription factors involved in T_RM_ regulation, most of them not only program T_RM_ tissue residency, but also have an effect on T_RM_ activation, metabolism, maintenance and homeostasis [13, 81, 82, 83, 84, 85, 86, 87, 88, 89, 90]. In this part, the transcription factors were summarized into four categories: activation, metabolism, maintenance and homeostasis, and introduced their effect on T_RM_ (Figure 4) (Table 2).

### 4.1 Transcriptional Regulation of T_RM_ Activation

Runx3 [81, 82], Blimp‐1 [84, 85], Hobit [84, 86] and AhR [87] control T_RM_ retention and cytotoxicity. In mouse models, Runx3 drives TGF‐β‐dependent CD69^+^CD103^+^ differentiation [81], in human T_RM_ cells, RUNX3 up‐regulates CD49a with RUNX2, and sustains granzyme B and perforin [82]. In mouse models, Blimp‐1 and Hobit repress *S1pr1*, *Sell* and *Ccr7* to block egress [83, 85]; Blimp‐1 induces granzyme B, while Hobit maintains granzyme expression [84]. AhR enhances T_RM_ identity by activating *Prdm1*, *Itgae* and *Gzmb* and repressing *Klf2*, *S1pr5* and *Klrg1* [87].

### 4.2 Transcriptional Regulation of T_RM_ Metabolism

T_RM_ activation is coupled to local metabolite availability, which shapes their metabolic state and function [96]. In a mouse model, Bhlhe40 sustains this link by promoting oxidative phosphorylation, TCA cycle, and respiratory chain gene expression, thereby maintaining mitochondrial fitness and acetyl‐CoA–dependent histone acetylation at the *Ifng*, *Gzmb* and *Itgae* loci [92]. Consequently, Bhlhe40‐deficient T_RM_ show reduced OXPHOS, downregulated CD69 and CD103, and impaired tissue retention [92].

### 4.3 Transcriptional Regulation of T_RM_ Maintenance

T‐bet [13] and Eomes [3, 86] have both been implicated in the maintenance of T_RM_ cells. Although their expression is typically downregulated during T_RM_ formation and their enforced expression can inhibit T_RM_ development, residual T‐bet expression is necessary for IL‐15‐mediated T_RM_ survival [13]. In the small intestine, Eomes promotes the expression of the anti‐apoptotic regulator Bcl‐2, thereby supporting the maintenance of established T_RM_ cells [3].

### 4.4 Transcriptional Regulation of T_RM_ Homeostasis

Dysregulation of T_RM_ homeostasis is associated with inflammatory and autoimmune conditions such as arthritis [97], allergies [98] and psoriasis [99], underscoring the importance of balanced T‐cell function. In a mouse model, NR4A1 [94, 100] is highly expressed in a quiescent T_RM_ subset marked by ABC transporters. Genetic deletion of these transporters leads to T_RM_ loss and an inflammatory, IFN‐γ‐high phenotype, suggesting that NR4A1 and ABC transporters cooperatively maintain T_RM_ homeostasis and quiescence [94]. Within the mouse small intestinal epithelium, Id2 and Id3 cooperatively maintain the long‐lived CD127^hi^ CD8^+^ T_RM_ pool; combined loss of Id2 and Id3 collapses the CD127^hi^ compartment and drives a subset of CD8^+^ T_RM_ to acquire a pro‐inflammatory phenotype [88]. Disruption of this axis thus impairs tissue retention and accelerates T_RM_ attrition. However, under chronic antigen stimulation murine NR4A proteins up‐regulate PD‐1 and TIM3 while suppressing IFN‐γ and TNF expression in CD8^+^ T cells [100], raising the possibility that the transcription factors governing T_RM_ homeostasis may simultaneously restrain inflammation and dampen antitumor effector function. These transcription factors, such as Blimp‐1 and Id3 provide a plausible mechanistic framework for the observed cytotoxic heterogeneity or functional differences of human T_RM_ cells, although additional regulators likely coexist.

## 5 Harnessing T_RM_ for Cancer Immunotherapy

**FIGURE 5:**
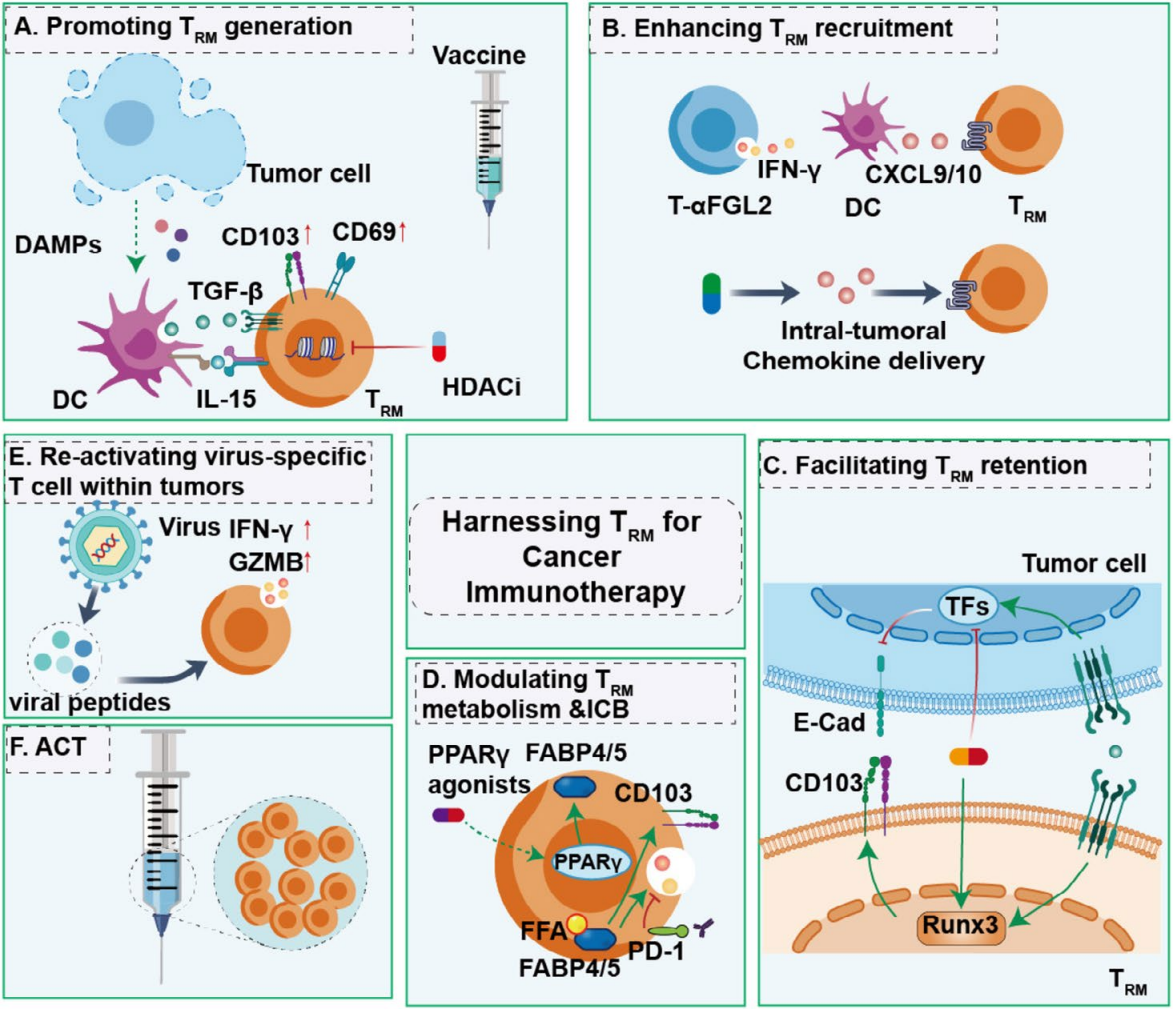
The strategies to enhance T_RM_ immunity. (A) Promoting T_RM_ generation and survival. (B) Enhancing T_RM_ recruitment to tumors. (C) Facilitating T_RM_ retention/adhesion. (D) Modulating metabolism and blocking immune checkpoints. (E) Re‐activating virus‐specific T cells within tumors. (F) Adoptive T_RM_ transfer.

T_RM_ cells are pivotal for long‐term immunity, and their presence in tumors correlates strongly with improved patient survival [2]. Harnessing these cells represents a promising frontier in cancer immunotherapy [9, 14]. This section delineates the core strategies to orchestrate potent T_RM_‐mediated anti‐tumor immunity, focusing on their generation, recruitment, retention, and reactivation within tumors (Figure 5).

### 5.1 Promoting T_RM_ Generation and Survival

Recent studies have demonstrated that specific immunization strategies and dendritic cell‐mediated signals, which initiate adaptive immune responses, can effectively promote the generation of T_RM_ [101, 102, 103]. Nizard et al. [101] showed that intranasal administration of a cancer vaccine composed of the B subunit of Shiga toxin (STxB) fused to an E7‐derived peptide from HPV16 effectively induced T_RM_ in the lung mucosa. Shin et al. [102] found that antigen‐specific activation of CD301b^+^ DCs plays a key role in activating T_RM_‐mediated protective immunity against genital HSV‐2 infection. Bourdely et al. [103] identified CD1c^+^CD163^+^ DCs as potent inducers of CD103^+^CD8^+^ T cells with T_RM_‐like features, in a TGF‐β‐dependent manner, and showed their association with improved antitumor responses in breast cancer. Signaling molecules such as IL‐12, IL‐15, and CD24, provided by CD8α^+^ and CD103^+^ DCs, have been shown to promote the priming of T_RM_ precursors during viral infection [46]. Recent studies have also shown that induction of immunogenic cell death (ICD) and/or pyroptosis in tumor cells effectively promotes DC maturation and initiates adaptive immune responses [104], raising the possibility that these mechanisms may also contribute to T_RM_ differentiation.

Epigenetic drugs such as HDAC inhibitors can promote T_RM_ differentiation [92]. Combining Tubastatin A (an HDAC6/8‐selective inhibitor) with acetate increases H3K27ac signal, markedly up‐regulates core T_RM_ markers including CD103 and CD69, and enhances the tissue‐residency capacity of virus‐ or tumor‐specific CD8^+^ T cells [92]. The HDAC3‐specific inhibitor RGFP966 rapidly upregulates granzyme B and perforin expression in activated CD8^+^ T cells, correlating with enhanced Runx3 and Blimp‐1 expression and immediate cytotoxic activity [105]; Runx3 and Blimp‐1 have also been implicated in T_RM_ regulation [81, 88, 106]. However, HDACi carry potential risks: prolonged or excessive HDAC3 inhibition can promote Blimp‐1‐mediated terminal effector differentiation, reduce the memory‐precursor pool, and increase post‐activation apoptosis, thereby compromising long‐term T_RM_ persistence and recall responses [88, 105]. High‐dose pan‐HDAC inhibitors can disrupt global histone‐acetylation homeostasis, provoking multi‐organ metabolic stress and transcriptional dysregulation [107, 108]. Whether HDACi can be exploited to boost T_RM_ function therefore hinges on compound selectivity, dosing, route of administration, and the specific immune‐microenvironment context; a closer‐to‐clinic model is still required to balance their pro‐effector versus pro‐exhaustion effects.

IL‐15 is a critical cytokine for the survival and functional activation of T_RM_ cells [2]. IL‐15 signaling via its receptor component CD122 is essential for maintaining T_RM_ pools in peripheral tissues such as the skin and lung [6, 13]. Notably, T_RM_ cells that lack T‐bet, which is necessary for CD122 expression, fail to persist long term, highlighting that T_RM_ survival depends on IL‐15‐mediated signaling [13]. In human tissues, IL‐15 also enhances the expression of cytotoxic molecules like perforin and granzyme B in T_RM_ cells, promoting their effector function upon stimulation [12]. These findings highlight IL‐15 not only as a survival factor but also as a functional activator of T_RM_ cells in tissue‐specific immune surveillance [109].

Engineering cytokines for targeted and sustained delivery has emerged as a viable strategy to enhance T_RM_ function while minimizing systemic toxicity [110]. Recent advances in cytokine engineering, such as fusion with collagen‐binding domains (CBD), allow for localized retention and gradual release of cytokines like IL‐7 and IL‐12 within the tumor microenvironment [110]. This approach significantly improves therapeutic efficacy and reduces immune‐related adverse events compared to systemic administration of native cytokines [110]. Given the potent effects of IL‐15 on T_RM_ biology, engineering IL‐15 for tissue‐targeted delivery may represents a promising avenue to enhance T_RM_‐mediated immunity in both infectious and oncological settings.

### 5.2 Enhancing T_RM_ Recruitment to Tumors

Harnessing chemokine receptors to enhance T_RM_ infiltration represents a promising strategy for bolstering antitumor immunity across multiple tissue contexts [33]. In ovarian cancer, CXCR6 is highly expressed on T_RM_‐like cells, and its ligand CXCL16, primarily produced by EpCAM^−^CD45^−^ stromal cells, facilitates their spatial retention within the tumor microenvironment, suggesting that CXCL16 delivery or CXCR6 engineering may promote T_RM_ accumulation [33]. Similarly, the CXCL17‐CXCR8 axis has been shown to recruit CD8^+^ T_EM_ and T_RM_ cells to the vaginal mucosa during herpes infection, highlighting its tissue‐specific mobilization potential [35]. In the skin, CXCR3 guides T_RM_ precursor entry into the epidermis following infection, facilitating their subsequent differentiation into long‐lived CD103^+^CD8^+^ T_RM_ cells [6].

Building on these mechanistic insights, therapeutic strategies can be designed to recruit and retain T_RM_ cells within tumors [111]. Zhao et al. [111] demonstrated that engineering T cells to block the immunosuppressive molecule FGL2 (T‐αFGL2) leads to robust induction of CXCL9 and CXCL10 in the glioblastoma microenvironment. This, in turn, recruits CXCR3^+^CD69^+^ CD8^+^ T cells, fostering the formation of tumor‐specific, brain‐resident T_RM_ cells with a clonally expanded TCR repertoire. These T_RM_ cells persist long‐term and confer durable antitumor immunity upon rechallenge, illustrating how microenvironment remodeling via engineered T cells can be leveraged to program T_RM_‐mediated tumor control [111].

While chemokine axes provide a molecular address code for T_RM_ recruitment, their effective delivery and local bioavailability remain the immediate bottleneck [112]. First, systemic administration of recombinant chemokines or encoding viruses may risks off‐target sequestration and rapid proteolytic clearance, blunting intratumoral gradients required for directional T‐cell migration [113, 114, 115]. Second, immune‐excluded or hypoxic lesions often display silencing of chemokines signal, thus, priming interventions, such as STING agonists, low‐dose irradiation, or oncolytic viruses, are needed to unlock endogenous chemokine transcription before T_RM_ infusion [116]. Third, stromal barriers (dense collagen, hyaluronan, aberrant vasculature) can physically obstruct gradient diffusion and T‐cell extravasation, necessitating transient ECM modulation or vessel‐normalizing agents to widen the recruitment window [117]. Finally, inter‐patient heterogeneity in baseline chemokine profiles implies that pretreatment biopsy‐guided stratification will be essential to identify individuals whose tumors can generate sufficient chemotactic signals, ensuring that subsequent T_RM_‐targeting therapies are applied only when recruitment is mechanistically feasible [112].

### 5.3 Facilitating T_RM_ Retention in the Tumor Microenvironment

CD44, CD49a, and CD103 act as “anchors” that promote the retention of T_RM_ cells in tissues by binding to their respective ligands, hyaluronic acid, collagen, and E‐cadherin [8]. These interactions ensure that T_RM_ cells remain firmly attached to the tissue, enabling them to provide rapid and localized immune surveillance against infections and tumors [8, 11]. Consistently, blockade of CD49a or CD103 has been shown to reduce CD8^+^ T cell infiltration and impair tumor control [29, 62].

The interaction between CD103 and its ligand E‐cadherin has gained attention in recent research [118]. Shields et al. [118] have yielded key insights into the understanding of how checkpoint blockade therapy works in the treatment of melanoma. They found that decreased expression of the protein E‐Cadherin in the tumors of melanoma patients seemed to inhibit the antitumor activity of CD103^+^ immune cells [118]. Consequently, this inhibited the overall responsiveness of checkpoint blockade therapy in melanoma patients with decreased levels of E‐Cadherin [118]. Conversely, in CD103‐knockout mouse models, the efficacy of checkpoint blockade was also diminished in E‐cadherin‐positive melanomas [118]. Corgnac et al. [119] further demonstrated that cancer stem cells (CSCs) can evade CD8^+^CD103^+^T_RM_‐like cell‐mediated cytotoxicity by downregulating E‐cadherin expression. Together, these studies highlight the critical role of E‐cadherin in enabling CD103^+^ T cell antitumor activity.

TGF‐β is a major factor in the downregulation of E‐Cadherin in the tumor microenvironment [120]. While it can induce CD103 expression [9], it can also decrease E‐Cadherin expression [120]. Given these two opposing effects, it is clear that attempting to achieve CD103 expression through TGF administration is not a viable solution. To address this issue, it is essential to find alternate strategies that are effective in inducing CD103 expression while preventing E‐Cadherin downregulation. To this end, it might be possible to intervene in the TGF‐β signaling pathway by inhibiting Slug [121] and PAK4 [122] to prevent E‐Cadherin downregulation, and by stimulating the downstream molecules of TGF‐β signaling in CD103 induction, such as Runx3 [106].

### 5.4 Modulating T_RM_ Metabolism for Enhanced Anti‐Tumor Immunity

T_RM_ cells exhibit distinct metabolic profiles compared to their circulating counterparts, a difference regulated by specific metabolic mediators [37]. In the presence of exogenous free fatty acids (FFAs), FABP4/FABP5 expression in CD8^+^ T_RM_ cells enhances mitochondrial oxidative metabolism, a response absent in *Fabp4*/*Fabp5* double‐knockout T_RM_ cells in murine models, Fabp4/Fabp5 deficiency results in impaired T_RM_ function and maintenance [37, 38].

The expression of FABP4 and FABP5 in T_RM_ cells is transcriptionally regulated by peroxisome proliferator‐activated receptor gamma (PPARγ); genetic knockdown or pharmacological inhibition of PPARγ significantly reduces FABP4/5 expression, impairing T_RM_ lipid uptake and survival [37].

Independently, PPAR agonists such as bezafibrate enhance fatty acid oxidation (FAO) in CD8^+^ T cells, promoting mitochondrial biogenesis and survival, which in turn boosts antitumor immunity and synergizes with anti‐PD‐1 therapy [123]. However, whether pharmacological activation of PPARγ, via bezafibrate or other agonists, can directly upregulate FABP4/5 expression and thereby enhance T_RM_ metabolic fitness remains to be experimentally tested. Thus, future studies should explicitly address the causal link between PPARγ activation and FABP4/5 abundance in T_RM_ cells. If confirmed, metabolic interventions that enhance FFA utilization and activate PPAR signaling could represent a rational strategy to bolster T_RM_‐mediated antitumor immunity.

Moreover, tumor cells can outcompete T_RM_‐like cells for lipid uptake, leading to T_RM_‐like cells' apoptosis; however, PD‐L1 blockade can reverse this metabolic competition and restore T_RM_‐like cells' survival and function [38].

### 5.5 Targeting Immune Checkpoints on T_RM_

Immune checkpoint molecules are an integral part of T_RM_/T_RM_‐like cells regulation, playing a dual role in the control of anti‐tumor immunity [7]. On the one hand, these molecules are capable of restraining T_RM_ activity in healthy tissue; on the other hand, they can also be used to weaken the immune response and reduce the activity of these cells against tumors [11, 14]. Some of the molecules involved in this process are PD‐1 and TIM3 [7]. T_RM_/T_RM_‐like cells have been observed in gastric cancer [67], melanoma [74] and breast cancer [7] and patients showed improved immunity after treatment with immune checkpoint inhibitors, associated with a positive response to ICB therapy. T_RM_‐like cells undergo functional reprogramming and clonal expansion during ICB [10, 77].

However, despite their activation and expansion during ICB, it remains unclear whether T_RM_ cells directly respond to PD‐1 blockade or are indirectly mobilized through the reinvigoration of precursor populations [14]. As highlighted by Gavil et al. [14], PD‐1 expression on T_RM_ cells may not necessarily indicate functional exhaustion but may instead reflect a history of antigen encounter. Similarly, Enamorado et al. [55] showed that anti‐PD‐1 therapy enhances T_RM_ infiltration in tumors, potentially by promoting the differentiation of central memory T cells (T_CM_) into T_RM_, rather than by reversing exhaustion within the T_RM_ compartment itself. Thus, whether T_RM_ cells are direct targets of PD‐1/PD‐L1 blockade or act as secondary effectors downstream of ICB remains an open question, and functionally dissecting their role in ICB response will be critical for optimizing immunotherapeutic strategies.

### 5.6 Re‐Activating Virus‐Specific T Cell Within Tumors

Virus‐specific T cells are widely present across multiple solid tumors [124, 125, 126, 127]. Although they are not generated in response to tumor antigens, accumulating evidence indicates that such virus‐specific T cells retain the capacity to be reactivated within the immunosuppressive tumor microenvironment and to participate in effective antitumor immunity [127, 128].

Intratumoral administration of viral peptides that match the individual's infection history can efficiently reactivate these bystander T cells [127, 128]. Once triggered, the re‐energized T cells release large amounts of effector cytokines (IFN‐γ, TNF‐α) and cytotoxic molecules (granzyme B), while simultaneously initiating a “pseudo‐viral infection” signaling cascade. Concomitant flow cytometry and RNA‐seq analyses show elevated CD86 on CD103^+^ DCs and higher MHC‐I on tumor cells, indicating that T_RM_ reactivation may enhance antigen presentation and broad immune infiltration [127, 128].

Viral peptides are well‐defined, chemically synthesized, highly immunogenic, and applicable to large patient populations without the need for personalized tumor‐antigen identification; further research could be done to verify the potential of harnessing virus‐specific T reactivation to turn “cold tumors” into “hot tumors” [14].

### 5.7 Adoptive T_RM_ Transfer/Chimeric Antigen Receptor‐T_RM_ Cell Therapy for Cancer Immunotherapy

Adoptive T‐cell immunotherapy is emerging as a promising strategy for cancer treatment [129]. While chimeric antigen receptor (CAR)‐T cell therapy has achieved remarkable success in treating hematological malignancies, its efficacy against solid tumors remains limited, partly due to the inability of infused T cells to traffic to and persist within tumor tissues [129]. Therefore, strategies to endow therapeutic T cells with tissue‐resident properties are being actively investigated.

Recent studies have explored several approaches to generate T_RM_ cells via adoptive transfer [81]. The first involves genetic engineering of T cells to overexpress key transcription factors known to drive T_RM_ differentiation. For instance, Milner et al. [81] demonstrated that CD8^+^ T cells overexpressing Runx3, a transcription factor critical for tissue residency, efficiently differentiated into T_RM_ cells upon adoptive transfer and significantly delayed tumor growth and prolonged survival in a melanoma challenge model, although complete tumor rejection was not achieved. Similarly, Crowl et al. [22] showed that overexpression of Hic1, a tissue‐specific transcriptional repressor, promoted T_RM_ formation particularly in the small intestine, while in salivary glands T_RM_ numbers were reduced, highlighting the context‐dependent regulation of T_RM_ programs and the potential for tissue‐targeted engineering.

The second strategy leverages the intrinsic plasticity of T_CM_ [55]. Enamorado et al. [55] employed a “T_CM_‐to‐T_RM_ conversion” strategy to demonstrate durable protection afforded by T_RM_: OT‐I T_CM_ cells were first adoptively transferred, and mice were then challenged with rVACV‐OVA skin infection or B16‐OVA tumors. Thirty days later, the T_CM_ cells had differentiated into stably resident CD69^+^CD103^+^ OT‐I T_RM_. FTY720 treatment or parabiosis to sequester circulating T cells left these T_RM_ populations as the sole immune component; they significantly controlled subsequent B16‐OVA tumor growth but did not achieve complete tumor rejection. Collectively, these studies demonstrate that engineering T cells to possess T_RM_ differentiation potential can significantly enhance anti‐tumor immunity [55]. This principle also provides a compelling strategic direction for improving CAR‐T cell therapy, by creating CAR‐T cells that are similarly programmed to develop into tissue‐resident populations upon infusion.

However, it is important to note that Gabriely et al. [80] provided a critical cautionary insight: not all CD103^+^CD8^+^ T cells possess antitumor activity. Their study revealed that a subset of CD103^+^CD8^+^ T cells exhibit a tolerogenic phenotype, characterized by high expression of immune‐inhibitory molecules such as CTLA‐4, IL‐10, and PD‐L1, and low expression of effector cytokines like IFN‐γ, TNF‐α, and granzymes. In CD8‐deficient hosts, adoptive transfer of these T_RM_‐like cells promoted rather than inhibited tumor growth and suppressed CD8^+^ T‐cell proliferation in vitro, partly via the PD‐1/PD‐L1 axis [80]. Therefore, direct adoptive transfer of unselected CD103^+^CD8^+^ T cells may fail to achieve therapeutic efficacy, or even prove counterproductive, due to functional heterogeneity within T_RM_‐like populations [80].

## 6 Conclusion and Perspective

This review briefly introduced the fundamental characteristics of T_RM_, functional roles and phenotypic determinants of T_RM_ in tumor immunity, the transcriptional regulation of T_RM_, the strategies to enhanced T_RM_ immunity, as well as the treatment strategies that can be taken to enhance T_RM_ anti‐tumor immunity. T_RM_ are absolutely essential elements in the fight against cancer, quickly responding to immune therapy. However, there are still some questions that need to be addressed.

### 6.1 Heterogeneity of T_RM_ Across Different Cancer Types

The heterogeneity of T_RM_ across different cancer types underscores the need for personalized immunotherapy strategies [130]. While T_RM_ are typically seen as a formidable fighter, recent advance researches noticed that the anti‐tumor ability of T_RM_ is impaired by some immune inhibitory molecules and transcription factors, which can even result in T_RM_ displaying immunosuppressive characteristics in certain types of tumors [78, 131]. This suggests that various types of T_RM_ regulators, combined with the unique nature of a tumor's microenvironment, could produce drastically different outcomes, which has important implications for cancer treatment and research [130]. Therefore, there is need to understand the function and regulators of T_RM_ in the tumor microenvironment and strategies utilizing those molecules to promote tumor‐killing ability while avoiding the immunosuppressive factors should be developed.

### 6.2 The Markers for Discriminating Tumor‐Specific T_RM_ Should Be Investigated

T_RM_ can be generated after the invasion of pathogens, such as tumors and viruses, and can persist in peripheral tissues for a long time [2]. This means that different pathogen‐specific T_RM_ can be present in the same tissues. Therefore, how can we distinguish tumor‐specific T_RM_ from bystander T_RM_? Early research has pointed out that CXCL13 and CD39 can be used as markers to differentiate tumor‐specific T_RM_ [41, 71]. However, further studies are required to validate the presence of more accurate and efficient markers for discriminating tumor‐specific T_RM_.

By unraveling the mechanisms underlying T_RM_ cell biology and developing innovative therapeutic strategies, we can harness the full potential of T_RM_ to revolutionize cancer immunotherapy and improve patient outcomes.

## Author Contributions

**Yao Xiao:** conceptualization, investigation, funding acquisition, writing – original draft, writing – review and editing, methodology. **Qi‐Chao Yang:** investigation, writing – original draft, writing – review and editing. **Zhi‐Jun Sun:** conceptualization, investigation, funding acquisition, writing – original draft, writing – review and editing, methodology, project administration, supervision.

## Funding

This work was supported by National Natural Science Foundation of China 82403447 (Y.X.), 82472818 (Z.‐J.S.), the Fundamental Research Funds for the Central Universities (2042022dx0003, 2042023kf0150), and Hubei Province International Science and Technology Cooperation Project 2021EHB027 (Z.‐J.S.).

## Ethics Statement

This is a review article. Therefore, ethical approval is not required for the study.

## Conflicts of Interest

The authors declare no conflicts of interest.

## Data Availability

Data sharing not applicable to this article as no datasets were generated or analyzed during the current study.

## References

[1] S. N. Mueller and L. K. Mackay , “Tissue‐Resident Memory T Cells: Local Specialists in Immune Defence,” Nature Reviews. Immunology 16 (2016): 79–89, 10.1038/nri.2015.3. 26688350

[2] S. Yenyuwadee , J. L. Sanchez‐Trincado Lopez , R. Shah , P. C. Rosato , and V. A. Boussiotis , “The Evolving Role of Tissue‐Resident Memory T Cells in Infections and Cancer,” Science Advances 8 (2022): eabo5871, 10.1126/sciadv.abo5871. 35977028 PMC9385156

[3] Y. H. Lin , H. G. Duong , A. E. Limary , et al., “Small Intestine and Colon Tissue‐Resident Memory CD8(+) T Cells Exhibit Molecular Heterogeneity and Differential Dependence on Eomes,” Immunity 56 (2023): 207–223.e8, 10.1016/j.immuni.2022.12.007. 36580919 PMC9904390

[4] M. E. Snyder , M. O. Finlayson , T. J. Connors , et al., “Generation and Persistence of Human Tissue‐Resident Memory T Cells in Lung Transplantation,” Science Immunology 4 (2019): eaav5581, 10.1126/sciimmunol.aav5581. 30850393 PMC6435356

[5] T. Wu , Y. Hu , Y. T. Lee , et al., “Lung‐Resident Memory CD8 T Cells (TRM) are Indispensable for Optimal Cross‐Protection Against Pulmonary Virus Infection,” Journal of Leukocyte Biology 95 (2014): 215–224, 10.1189/jlb.0313180. 24006506 PMC3896663

[6] L. K. Mackay , A. Rahimpour , J. Z. Ma , et al., “The Developmental Pathway for CD103(+)CD8+ Tissue‐Resident Memory T Cells of Skin,” Nature Immunology 14 (2013): 1294–1301, 10.1038/ni.2744. 24162776

[7] A. Byrne , P. Savas , S. Sant , et al., “Tissue‐Resident Memory T Cells in Breast Cancer Control and Immunotherapy Responses,” Nature Reviews. Clinical Oncology 17 (2020): 341–348, 10.1038/s41571-020-0333-y. 32112054

[8] D. Amsen , K. van Gisbergen , P. Hombrink , and R. A. W. van Lier , “Tissue‐Resident Memory T Cells at the Center of Immunity to Solid Tumors,” Nature Immunology 19 (2018): 538–546, 10.1038/s41590-018-0114-2. 29777219

[9] K. Okla , D. L. Farber , and W. Zou , “Tissue‐Resident Memory T Cells in Tumor Immunity and Immunotherapy,” Journal of Experimental Medicine 218 (2021): e20201605, 10.1084/jem.20201605. 33755718 PMC7992502

[10] A. M. Luoma , S. Suo , Y. Wang , et al., “Tissue‐Resident Memory and Circulating T Cells Are Early Responders to Pre‐Surgical Cancer Immunotherapy,” Cell 185 (2022): 2918–2935.e29, 10.1016/j.cell.2022.06.018. 35803260 PMC9508682

[11] M. Buggert , D. A. Price , L. K. Mackay , and M. R. Betts , “Human Circulating and Tissue‐Resident Memory CD8(+) T Cells,” Nature Immunology 24 (2023): 1076–1086, 10.1038/s41590-023-01538-6. 37349380

[12] J. Niessl , T. R. Muller , C. Constantz , et al., “Tissue Origin and Virus Specificity Shape Human CD8(+) T Cell Cytotoxicity,” Science Immunology 10 (2025): eadq4881, 10.1126/sciimmunol.adq4881.40680144

[13] L. K. Mackay , E. Wynne‐Jones , D. Freestone , et al., “T‐Box Transcription Factors Combine With the Cytokines TGF‐Beta and IL‐15 to Control Tissue‐Resident Memory T Cell Fate,” Immunity 43 (2015): 1101–1111, 10.1016/j.immuni.2015.11.008. 26682984

[14] N. V. Gavil , K. Cheng , and D. Masopust , “Resident Memory T Cells and Cancer,” Immunity 57 (2024): 1734–1751, 10.1016/j.immuni.2024.06.017. 39142275 PMC11529779

[15] L. K. Beura , S. Wijeyesinghe , E. A. Thompson , et al., “T Cells in Nonlymphoid Tissues Give Rise to Lymph‐Node‐Resident Memory T Cells,” Immunity 48 (2018): 327–338.e5, 10.1016/j.immuni.2018.01.015. 29466758 PMC5828517

[16] H. Wang , N. V. Gavil , N. Koewler , D. Masopust , and S. C. Jameson , “Parabiosis in Mice to Study Tissue Residency of Immune Cells,” Current Protocols 2 (2022): e446, 10.1002/cpz1.446. 35612420 PMC9216177

[17] D. Masopust , D. Choo , V. Vezys , et al., “Dynamic T Cell Migration Program Provides Resident Memory Within Intestinal Epithelium,” Journal of Experimental Medicine 207 (2010): 553–564, 10.1084/jem.20090858. 20156972 PMC2839151

[18] S. R. Schwab and J. G. Cyster , “Finding a Way out: Lymphocyte Egress From Lymphoid Organs,” Nature Immunology 8 (2007): 1295–1301, 10.1038/ni1545. 18026082

[19] P. A. Szabo , M. Miron , and D. L. Farber , “Location, Location, Location: Tissue Resident Memory T Cells in Mice and Humans,” Science Immunology 4 (2019), 10.1126/sciimmunol.aas9673. PMC6778482 30952804

[20] K. G. Anderson , K. Mayer‐Barber , H. Sung , et al., “Intravascular Staining for Discrimination of Vascular and Tissue Leukocytes,” Nature Protocols 9 (2014): 209–222, 10.1038/nprot.2014.005. 24385150 PMC4428344

[21] R. A. Clark , R. Watanabe , J. E. Teague , et al., “Skin Effector Memory T Cells Do Not Recirculate and Provide Immune Protection in Alemtuzumab‐Treated CTCL Patients,” Science Translational Medicine 4 (2012): 117ra7, 10.1126/scitranslmed.3003008. PMC3373186 22261031

[22] J. T. Crowl , M. Heeg , A. Ferry , et al., “Tissue‐Resident Memory CD8(+) T Cells Possess Unique Transcriptional, Epigenetic and Functional Adaptations to Different Tissue Environments,” Nature Immunology 23 (2022): 1121–1131, 10.1038/s41590-022-01229-8. 35761084 PMC10041538

[23] Y. Koda , T. Teratani , P. S. Chu , et al., “CD8(+) Tissue‐Resident Memory T Cells Promote Liver Fibrosis Resolution by Inducing Apoptosis of Hepatic Stellate Cells,” Nature Communications 12 (2021): 4474, 10.1038/s41467-021-24734-0. PMC8298513 34294714

[24] D. Cibrian and F. Sanchez‐Madrid , “CD69: From Activation Marker to Metabolic Gatekeeper,” European Journal of Immunology 47 (2017): 946–953, 10.1002/eji.201646837. 28475283 PMC6485631

[25] D. A. Walsh , H. Borges da Silva , L. K. Beura , et al., “The Functional Requirement for CD69 in Establishment of Resident Memory CD8(+) T Cells Varies With Tissue Location,” Journal of Immunology 203 (2019): 946–955, 10.4049/jimmunol.1900052. PMC6684481 31243092

[26] E. C. Reilly , K. Lambert Emo , P. M. Buckley , et al., “T(RM) Integrins CD103 and CD49a Differentially Support Adherence and Motility After Resolution of Influenza Virus Infection,” Proceedings of the National Academy of Sciences of the United States of America 117 (2020): 12306–12314, 10.1073/pnas.1915681117. 32439709 PMC7275699

[27] M. Abd Hamid , H. Colin‐York , N. Khalid‐Alham , et al., “Self‐Maintaining CD103(+) Cancer‐Specific T Cells Are Highly Energetic With Rapid Cytotoxic and Effector Responses,” Cancer Immunology Research 8 (2020): 203–216, 10.1158/2326-6066.CIR-19-0554. 31771983 PMC7611226

[28] S. Cheuk , H. Schlums , I. Gallais Serezal , et al., “CD49a Expression Defines Tissue‐Resident CD8(+) T Cells Poised for Cytotoxic Function in Human Skin,” Immunity 46 (2017): 287–300, 10.1016/j.immuni.2017.01.009. 28214226 PMC5337619

[29] F. Sandoval , M. Terme , M. Nizard , et al., “Mucosal Imprinting of Vaccine‐Induced CD8(+) T Cells Is Crucial to Inhibit the Growth of Mucosal Tumors,” Science Translational Medicine 5 (2013): 172ra20, 10.1126/scitranslmed.3004888. PMC4086646 23408053

[30] S. J. Ray , S. N. Franki , R. H. Pierce , et al., “The Collagen Binding alpha1beta1 Integrin VLA‐1 Regulates CD8 T Cell‐Mediated Immune Protection Against Heterologous Influenza Infection,” Immunity 20 (2004): 167–179, 10.1016/s1074-7613(04)00021-4. 14975239

[31] C. N. Skon , J. Y. Lee , K. G. Anderson , D. Masopust , K. A. Hogquist , and S. C. Jameson , “Transcriptional Downregulation of S1pr1 Is Required for the Establishment of Resident Memory CD8+ T Cells,” Nature Immunology 14 (2013): 1285–1293, 10.1038/ni.2745. 24162775 PMC3844557

[32] L. K. Mackay , A. Braun , B. L. Macleod , et al., “Cutting Edge: CD69 Interference With Sphingosine‐1‐Phosphate Receptor Function Regulates Peripheral T Cell Retention,” Journal of Immunology 194 (2015): 2059–2063, 10.4049/jimmunol.1402256. 25624457

[33] R. Muthuswamy , A. R. McGray , S. Battaglia , et al., “CXCR6 by Increasing Retention of Memory CD8(+) T Cells in the Ovarian Tumor Microenvironment Promotes Immunosurveillance and Control of Ovarian Cancer,” Journal for Immunotherapy of Cancer 9 (2021): e003329, 10.1136/jitc-2021-003329. 34607898 PMC8491420

[34] A. Zaid , J. L. Hor , S. N. Christo , et al., “Chemokine Receptor‐Dependent Control of Skin Tissue‐Resident Memory T Cell Formation,” Journal of Immunology 199 (2017): 2451–2459, 10.4049/jimmunol.1700571. 28855310

[35] R. Srivastava , M. Hernandez‐Ruiz , A. A. Khan , et al., “CXCL17 Chemokine‐Dependent Mobilization of CXCR8(+)CD8(+) Effector Memory and Tissue‐Resident Memory T Cells in the Vaginal Mucosa Is Associated With Protection Against Genital Herpes,” Journal of Immunology 200 (2018): 2915–2926, 10.4049/jimmunol.1701474. PMC5893430 29549178

[36] S. Caldeira‐Dantas , T. Furmanak , C. Smith , et al., “The Chemokine Receptor CXCR3 Promotes CD8(+) T Cell Accumulation in Uninfected Salivary Glands but Is Not Necessary After Murine Cytomegalovirus Infection,” Journal of Immunology 200 (2018): 1133–1145, 10.4049/jimmunol.1701272. PMC5780235 29288198

[37] Y. Pan , T. Tian , C. O. Park , et al., “Survival of Tissue‐Resident Memory T Cells Requires Exogenous Lipid Uptake and Metabolism,” Nature 543 (2017): 252–256, 10.1038/nature21379. 28219080 PMC5509051

[38] R. Lin , H. Zhang , Y. Yuan , et al., “Fatty Acid Oxidation Controls CD8(+) Tissue‐Resident Memory T‐Cell Survival in Gastric Adenocarcinoma,” Cancer Immunology Research 8 (2020): 479–492, 10.1158/2326-6066.CIR-19-0702. 32075801

[39] N. V. Gavil , M. C. Scott , E. Weyu , et al., “Chronic Antigen in Solid Tumors Drives a Distinct Program of T Cell Residence,” Science Immunology 8 (2023): eadd5976, 10.1126/sciimmunol.add5976. 37267383 PMC10569081

[40] Y. J. Lee , J. Y. Kim , S. H. Jeon , et al., “CD39(+) Tissue‐Resident Memory CD8(+) T Cells With a Clonal Overlap Across Compartments Mediate Antitumor Immunity in Breast Cancer,” Science Immunology 7 (2022): eabn8390, 10.1126/sciimmunol.abn8390. 36026440

[41] T. Duhen , R. Duhen , R. Montler , et al., “Co‐Expression of CD39 and CD103 Identifies Tumor‐Reactive CD8 T Cells in Human Solid Tumors,” Nature Communications 9 (2018): 2724, 10.1038/s41467-018-05072-0. PMC6045647 30006565

[42] A. Chow , F. Z. Uddin , M. Liu , et al., “The Ectonucleotidase CD39 Identifies Tumor‐Reactive CD8(+) T Cells Predictive of Immune Checkpoint Blockade Efficacy in Human Lung Cancer,” Immunity 56 (2023): 93–106.e6, 10.1016/j.immuni.2022.12.001. 36574773 PMC9887636

[43] A. Losurdo , C. Scirgolea , G. Alvisi , et al., “Single‐Cell Profiling Defines the Prognostic Benefit of CD39(High) Tissue Resident Memory CD8+ T Cells in Luminal‐Like Breast Cancer,” Communications Biology 4 (2021): 1117, 10.1038/s42003-021-02595-z. 34552178 PMC8458450

[44] L. M. Wakim , J. Smith , I. Caminschi , M. H. Lahoud , and J. A. Villadangos , “Antibody‐Targeted Vaccination to Lung Dendritic Cells Generates Tissue‐Resident Memory CD8 T Cells That Are Highly Protective Against Influenza Virus Infection,” Mucosal Immunology 8 (2015): 1060–1071, 10.1038/mi.2014.133. 25586557

[45] V. Mani , S. K. Bromley , T. Aijo , et al., “Migratory DCs Activate TGF‐Beta to Precondition Naive CD8(+) T Cells for Tissue‐Resident Memory Fate,” Science 366 (2019): eaav5728, 10.1126/science.aav5728. 31601741 PMC6939608

[46] S. Iborra , M. Martinez‐Lopez , S. C. Khouili , et al., “Optimal Generation of Tissue‐Resident but Not Circulating Memory T Cells During Viral Infection Requires Crosspriming by DNGR‐1(+) Dendritic Cells,” Immunity 45 (2016): 847–860, 10.1016/j.immuni.2016.08.019. 27692611 PMC5074364

[47] T. Adachi , T. Kobayashi , E. Sugihara , et al., “Hair Follicle‐Derived IL‐7 and IL‐15 Mediate Skin‐Resident Memory T Cell Homeostasis and Lymphoma,” Nature Medicine 21 (2015): 1272–1279, 10.1038/nm.3962. PMC4636445 26479922

[48] S. K. Bromley , H. Akbaba , V. Mani , et al., “CD49a Regulates Cutaneous Resident Memory CD8(+) T Cell Persistence and Response,” Cell Reports 32 (2020): 108085, 10.1016/j.celrep.2020.108085. 32877667 PMC7520726

[49] L. Kok , D. Masopust , and T. N. Schumacher , “The Precursors of CD8(+) Tissue Resident Memory T Cells: From Lymphoid Organs to Infected Tissues,” Nature Reviews. Immunology 22 (2022): 283–293, 10.1038/s41577-021-00590-3. PMC8415193 34480118

[50] L. Kok , F. E. Dijkgraaf , J. Urbanus , et al., “A Committed Tissue‐Resident Memory T Cell Precursor Within the Circulating CD8+ Effector T Cell Pool,” Journal of Experimental Medicine 217 (2020), 10.1084/jem.20191711. PMC7537386 32728699

[51] J. R. Webb , K. Milne , P. Watson , R. J. Deleeuw , and B. H. Nelson , “Tumor‐Infiltrating Lymphocytes Expressing the Tissue Resident Memory Marker CD103 Are Associated With Increased Survival in High‐Grade Serous Ovarian Cancer,” Clinical Cancer Research 20 (2014): 434–444, 10.1158/1078-0432.CCR-13-1877. 24190978

[52] S. L. Park , A. Buzzai , J. Rautela , et al., “Tissue‐Resident Memory CD8(+) T Cells Promote Melanoma‐Immune Equilibrium in Skin,” Nature 565 (2019): 366–371, 10.1038/s41586-018-0812-9. 30598548

[53] M. Roerden and S. Spranger , “Cancer Immune Evasion, Immunoediting and Intratumour Heterogeneity,” Nature Reviews. Immunology 25 (2025): 353–369, 10.1038/s41577-024-01111-8. 39748116

[54] F. Galvez‐Cancino , E. Lopez , E. Menares , et al., “Vaccination‐Induced Skin‐Resident Memory CD8(+) T Cells Mediate Strong Protection Against Cutaneous Melanoma,” Oncoimmunology 7 (2018): e1442163, 10.1080/2162402X.2018.1442163. 29900048 PMC5993487

[55] M. Enamorado , S. Iborra , E. Priego , et al., “Enhanced Anti‐Tumour Immunity Requires the Interplay Between Resident and Circulating Memory CD8(+) T Cells,” Nature Communications 8 (2017): 16073, 10.1038/ncomms16073. PMC5520051 28714465

[56] B. T. Malik , K. T. Byrne , J. L. Vella , et al., “Resident Memory T Cells in the Skin Mediate Durable Immunity to Melanoma,” Science Immunology 2 (2017), 10.1126/sciimmunol.aam6346. PMC5525335 28738020

[57] A. K. Molodtsov , N. Khatwani , J. L. Vella , et al., “Resident Memory CD8(+) T Cells in Regional Lymph Nodes Mediate Immunity to Metastatic Melanoma,” Immunity 54 (2021): 2117–2132.e7, 10.1016/j.immuni.2021.08.019. 34525340 PMC9015193

[58] F. Mami‐Chouaib , C. Blanc , S. Corgnac , et al., “Resident Memory T Cells, Critical Components in Tumor Immunology,” Journal for ImmunoTherapy of Cancer 6 (2018): 87, 10.1186/s40425-018-0399-6. 30180905 PMC6122734

[59] M. Evrard , E. Becht , R. Fonseca , et al., “Single‐Cell Protein Expression Profiling Resolves Circulating and Resident Memory T Cell Diversity Across Tissues and Infection Contexts,” Immunity 56 (2023): 1664–1680.e9, 10.1016/j.immuni.2023.06.005. 37392736

[60] W. Su , J. Saravia , I. Risch , et al., “CXCR6 Orchestrates Brain CD8(+) T Cell Residency and Limits Mouse Alzheimer's Disease Pathology,” Nature Immunology 24 (2023): 1735–1747, 10.1038/s41590-023-01604-z. 37679549 PMC11102766

[61] F. Djenidi , J. Adam , A. Goubar , et al., “CD8+CD103+ Tumor‐Infiltrating Lymphocytes Are Tumor‐Specific Tissue‐Resident Memory T Cells and a Prognostic Factor for Survival in Lung Cancer Patients,” Journal of Immunology 194 (2015): 3475–3486, 10.4049/jimmunol.1402711. 25725111

[62] Y. Xiao , L. Mao , Q. C. Yang , et al., “CD103 Blockade Impair Anti‐CTLA‐4 Immunotherapy in Oral Cancer,” Oral Oncology 138 (2023): 106331, 10.1016/j.oraloncology.2023.106331. 36753904

[63] C. M. Anadon , X. Yu , K. Hanggi , et al., “Ovarian Cancer Immunogenicity Is Governed by a Narrow Subset of Progenitor Tissue‐Resident Memory T Cells,” Cancer Cell 40 (2022): 545–557.e13, 10.1016/j.ccell.2022.03.008. 35427494 PMC9096229

[64] Y. Xiao , H. Li , L. Mao , et al., “CD103(+) T and Dendritic Cells Indicate a Favorable Prognosis in Oral Cancer,” Journal of Dental Research 98 (2019): 1480–1487, 10.1177/0022034519882618. 31658426

[65] M. M. Melssen , R. S. Lindsay , K. Stasiak , et al., “Differential Expression of CD49a and CD49b Determines Localization and Function of Tumor‐Infiltrating CD8(+) T Cells,” Cancer Immunology Research 9 (2021): 583–597, 10.1158/2326-6066.CIR-20-0427. 33619119 PMC8102369

[66] A. Le Floc'h , A. Jalil , K. Franciszkiewicz , P. Validire , I. Vergnon , and F. Mami‐Chouaib , “Minimal Engagement of CD103 on Cytotoxic T Lymphocytes With an E‐Cadherin‐Fc Molecule Triggers Lytic Granule Polarization via a Phospholipase Cgamma‐Dependent Pathway,” Cancer Research 71 (2011): 328–338, 10.1158/0008-5472.CAN-10-2457. 21224355

[67] R. Li , H. Liu , Y. Cao , et al., “Identification and Validation of an Immunogenic Subtype of Gastric Cancer With Abundant Intratumoural CD103(+)CD8(+) T Cells Conferring Favourable Prognosis,” British Journal of Cancer 122 (2020): 1525–1534, 10.1038/s41416-020-0813-y. 32205862 PMC7217759

[68] V. P. Ma , Y. Hu , A. V. Kellner , et al., “The Magnitude of LFA‐1/ICAM‐1 Forces Fine‐Tune TCR‐Triggered T Cell Activation,” Science Advances 8 (2022): eabg4485, 10.1126/sciadv.abg4485. 35213231 PMC8880789

[69] E. Menares , F. Galvez‐Cancino , P. Caceres‐Morgado , et al., “Tissue‐Resident Memory CD8(+) T Cells Amplify Anti‐Tumor Immunity by Triggering Antigen Spreading Through Dendritic Cells,” Nature Communications 10 (2019): 4401, 10.1038/s41467-019-12319-x. PMC6765014 31562311

[70] J. M. Schenkel , K. A. Fraser , L. K. Beura , K. E. Pauken , V. Vezys , and D. Masopust , “T Cell Memory. Resident Memory CD8 T Cells Trigger Protective Innate and Adaptive Immune Responses,” Science 346 (2014): 98–101, 10.1126/science.1254536. 25170049 PMC4449618

[71] H. H. Workel , J. M. Lubbers , R. Arnold , et al., “A Transcriptionally Distinct CXCL13(+)CD103(+)CD8(+) T‐Cell Population Is Associated With B‐Cell Recruitment and Neoantigen Load in Human Cancer,” Cancer Immunology Research 7 (2019): 784–796, 10.1158/2326-6066.CIR-18-0517. 30872264

[72] C. Hu , W. You , D. Kong , et al., “Tertiary Lymphoid Structure‐Associated B Cells Enhance CXCL13(+)CD103(+)CD8(+) Tissue‐Resident Memory T‐Cell Response to Programmed Cell Death Protein 1 Blockade in Cancer Immunotherapy,” Gastroenterology 166 (2024): 1069–1084, 10.1053/j.gastro.2023.10.022. 38445519

[73] P. Savas , B. Virassamy , C. Ye , et al., “Single‐Cell Profiling of Breast Cancer T Cells Reveals a Tissue‐Resident Memory Subset Associated With Improved Prognosis,” Nature Medicine 24 (2018): 986–993, 10.1038/s41591-018-0078-7. 29942092

[74] J. Edwards , J. S. Wilmott , J. Madore , et al., “CD103(+) Tumor‐Resident CD8(+) T Cells Are Associated With Improved Survival in Immunotherapy‐Naive Melanoma Patients and Expand Significantly During Anti‐PD‐1 Treatment,” Clinical Cancer Research 24 (2018): 3036–3045, 10.1158/1078-0432.CCR-17-2257. 29599411

[75] S. Corgnac , I. Malenica , L. Mezquita , et al., “CD103(+)CD8(+) T(RM) Cells Accumulate in Tumors of Anti‐PD‐1‐Responder Lung Cancer Patients and Are Tumor‐Reactive Lymphocytes Enriched With Tc17,” Cell Reports Medicine 1 (2020): 100127, 10.1016/j.xcrm.2020.100127. 33205076 PMC7659589

[76] G. Oliveira , A. M. Egloff , A. B. Afeyan , et al., “Preexisting Tumor‐Resident T Cells With Cytotoxic Potential Associate With Response to Neoadjuvant Anti‐PD‐1 in Head and Neck Cancer,” Science Immunology 8 (2023): eadf4968, 10.1126/sciimmunol.adf4968. 37683037 PMC10794154

[77] J. Han , Y. Zhao , K. Shirai , et al., “Resident and Circulating Memory T Cells Persist for Years in Melanoma Patients With Durable Responses to Immunotherapy,” Nature Cancer 2 (2021): 300–311, 10.1038/s43018-021-00180-1. 34179824 PMC8223731

[78] C. Lai , G. Coltart , A. Shapanis , et al., “CD8+CD103+ Tissue‐Resident Memory T Cells Convey Reduced Protective Immunity in Cutaneous Squamous Cell Carcinoma,” Journal for ImmunoTherapy of Cancer 9 (2021): e001807, 10.1136/jitc-2020-001807. 33479027 PMC7825273

[79] C. Sanders , A. S. M. Hamad , S. Ng , et al., “CD103+ Tissue Resident T‐Lymphocytes Accumulate in Lung Metastases and Are Correlated With Poor Prognosis in ccRCC,” Cancers 14 (2022): 1541, 10.3390/cancers14061541. 35326691 PMC8946052

[80] G. Gabriely , A. P. da Cunha , R. M. Rezende , et al., “Targeting Latency‐Associated Peptide Promotes Antitumor Immunity,” Science Immunology 2 (2017): eaaj1738, 10.1126/sciimmunol.aaj1738. 28763794 PMC5657397

[81] J. J. Milner , C. Toma , B. Yu , et al., “Runx3 Programs CD8(+) T Cell Residency in Non‐Lymphoid Tissues and Tumours,” Nature 552 (2017): 253–257, 10.1038/nature24993. 29211713 PMC5747964

[82] B. Zitti , E. Hoffer , W. Zheng , et al., “Human Skin‐Resident CD8(+) T Cells Require RUNX2 and RUNX3 for Induction of Cytotoxicity and Expression of the Integrin CD49a,” Immunity 56 (2023): 1285–1302.e7, 10.1016/j.immuni.2023.05.003. 37269830

[83] L. K. Mackay , M. Minnich , N. A. Kragten , et al., “Hobit and Blimp1 Instruct a Universal Transcriptional Program of Tissue Residency in Lymphocytes,” Science 352 (2016): 459–463, 10.1126/science.aad2035. 27102484

[84] N. A. M. Kragten , F. M. Behr , F. A. Vieira Braga , et al., “Blimp‐1 Induces and Hobit Maintains the Cytotoxic Mediator Granzyme B in CD8 T Cells,” European Journal of Immunology 48 (2018): 1644–1662, 10.1002/eji.201847771. 30051906

[85] L. Parga‐Vidal , R. Taggenbrock , A. Beumer‐Chuwonpad , et al., “Hobit and Blimp‐1 Regulate T(RM) Abundance After LCMV Infection by Suppressing Tissue Exit Pathways of T(RM) Precursors,” European Journal of Immunology 52 (2022): 1095–1111, 10.1002/eji.202149665. 35389518 PMC9545210

[86] L. Parga‐Vidal , F. M. Behr , N. A. M. Kragten , et al., “Hobit Identifies Tissue‐Resident Memory T Cell Precursors That Are Regulated by Eomes,” Science Immunology 6 (2021): eabg3533, 10.1126/sciimmunol.abg3533. 34417257

[87] J. W. Dean , E. Y. Helm , Z. Fu , et al., “The Aryl Hydrocarbon Receptor Cell Intrinsically Promotes Resident Memory CD8(+) T Cell Differentiation and Function,” Cell Reports 42 (2023): 111963, 10.1016/j.celrep.2022.111963. 36640340 PMC9940759

[88] J. J. Milner , C. Toma , Z. He , et al., “Heterogenous Populations of Tissue‐Resident CD8(+) T Cells Are Generated in Response to Infection and Malignancy,” Immunity 52 (2020): 808–824.e7, 10.1016/j.immuni.2020.04.007. 32433949 PMC7784612

[89] X. Li , X. Yan , Y. Wang , B. Kaur , H. Han , and J. Yu , “The Notch Signaling Pathway: A Potential Target for Cancer Immunotherapy,” Journal of Hematology & Oncology 16 (2023): 45, 10.1186/s13045-023-01439-z. 37131214 PMC10155406

[90] P. Hombrink , C. Helbig , R. A. Backer , et al., “Programs for the Persistence, Vigilance and Control of Human CD8(+) Lung‐Resident Memory T Cells,” Nature Immunology 17 (2016): 1467–1478, 10.1038/ni.3589. 27776108

[91] A. Zaid , L. K. Mackay , A. Rahimpour , et al., “Persistence of Skin‐Resident Memory T Cells Within an Epidermal Niche,” Proceedings of the National Academy of Sciences of the United States of America 111 (2014): 5307–5312, 10.1073/pnas.1322292111. 24706879 PMC3986170

[92] C. Li , B. Zhu , Y. M. Son , et al., “The Transcription Factor Bhlhe40 Programs Mitochondrial Regulation of Resident CD8(+) T Cell Fitness and Functionality,” Immunity 51 (2019): 491–507.e7, 10.1016/j.immuni.2019.08.013. 31533057 PMC6903704

[93] M. Evrard , E. Wynne‐Jones , C. Peng , et al., “Sphingosine 1‐Phosphate Receptor 5 (S1PR5) Regulates the Peripheral Retention of Tissue‐Resident Lymphocytes,” Journal of Experimental Medicine 219 (2022): e20210116, 10.1084/jem.20210116. 34677611 PMC8546662

[94] C. S. Boddupalli , S. Nair , S. M. Gray , et al., “ABC Transporters and NR4A1 Identify a Quiescent Subset of Tissue‐Resident Memory T Cells,” Journal of Clinical Investigation 126 (2016): 3905–3916, 10.1172/JCI85329. 27617863 PMC5096804

[95] X. Liu , Y. Wang , H. Lu , et al., “Genome‐Wide Analysis Identifies NR4A1 as a Key Mediator of T Cell Dysfunction,” Nature 567 (2019): 525–529, 10.1038/s41586-019-0979-8. 30814730 PMC6507425

[96] S. Konjar , C. Ferreira , F. S. Carvalho , et al., “Intestinal Tissue‐Resident T Cell Activation Depends on Metabolite Availability,” Proceedings of the National Academy of Sciences of the United States of America 119 (2022): e2202144119, 10.1073/pnas.2202144119. 35969785 PMC9411733

[97] M. H. Chang , A. Levescot , N. Nelson‐Maney , et al., “Arthritis Flares Mediated by Tissue‐Resident Memory T Cells in the Joint,” Cell Reports 37 (2021): 109902, 10.1016/j.celrep.2021.109902. 34706228 PMC8561718

[98] A. B. Funch , V. Mraz , A. O. Gadsboll , et al., “CD8(+) Tissue‐Resident Memory T Cells Recruit Neutrophils That Are Essential for Flare‐Ups in Contact Dermatitis,” Allergy 77 (2022): 513–524, 10.1111/all.14986. 34169536

[99] L. Chen and Z. Shen , “Tissue‐Resident Memory T Cells and Their Biological Characteristics in the Recurrence of Inflammatory Skin Disorders,” Cellular & Molecular Immunology 17 (2020): 64–75, 10.1038/s41423-019-0291-4. 31595056 PMC6952397

[100] J. Chen , I. F. Lopez‐Moyado , H. Seo , et al., “NR4A Transcription Factors Limit CAR T Cell Function in Solid Tumours,” Nature 567 (2019): 530–534, 10.1038/s41586-019-0985-x. 30814732 PMC6546093

[101] M. Nizard , H. Roussel , M. O. Diniz , et al., “Induction of Resident Memory T Cells Enhances the Efficacy of Cancer Vaccine,” Nature Communications 8 (2017): 15221, 10.1038/ncomms15221. PMC5458068 28537262

[102] H. Shin , Y. Kumamoto , S. Gopinath , and A. Iwasaki , “CD301b+ Dendritic Cells Stimulate Tissue‐Resident Memory CD8+ T Cells to Protect Against Genital HSV‐2,” Nature Communications 7 (2016): 13346, 10.1038/ncomms13346. PMC5105190 27827367

[103] P. Bourdely , G. Anselmi , K. Vaivode , et al., “Transcriptional and Functional Analysis of CD1c(+) Human Dendritic Cells Identifies a CD163(+) Subset Priming CD8(+)CD103(+) T Cells,” Immunity 53 (2020): 335–352.e8, 10.1016/j.immuni.2020.06.002. 32610077 PMC7445430

[104] Y. Wu , J. Zhang , S. Yu , et al., “Cell Pyroptosis in Health and Inflammatory Diseases,” Cell Death Discovery 8 (2022): 191, 10.1038/s41420-022-00998-3. 35411030 PMC8995683

[105] R. E. Tay , O. Olawoyin , P. Cejas , et al., “Hdac3 Is an Epigenetic Inhibitor of the Cytotoxicity Program in CD8 T Cells,” Journal of Experimental Medicine 217 (2020): e20191453, 10.1084/jem.20191453. 32374402 PMC7336313

[106] R. Fonseca , T. N. Burn , L. C. Gandolfo , et al., “Runx3 Drives a CD8(+) T Cell Tissue Residency Program That Is Absent in CD4(+) T Cells,” Nature Immunology 23 (2022): 1236–1245, 10.1038/s41590-022-01273-4. 35882933 PMC13045866

[107] W. Dai , X. Qiao , Y. Fang , et al., “Epigenetics‐Targeted Drugs: Current Paradigms and Future Challenges,” Signal Transduction and Targeted Therapy 9 (2024): 332, 10.1038/s41392-024-02039-0. 39592582 PMC11627502

[108] J. Song , P. Yang , C. Chen , et al., “Targeting Epigenetic Regulators as a Promising Avenue to Overcome Cancer Therapy Resistance,” Signal Transduction and Targeted Therapy 10 (2025): 219, 10.1038/s41392-025-02266-z. 40675967 PMC12271501

[109] B. Jabri and V. Abadie , “IL‐15 Functions as a Danger Signal to Regulate Tissue‐Resident T Cells and Tissue Destruction,” Nature Reviews. Immunology 15 (2015): 771–783, 10.1038/nri3919. PMC5079184 26567920

[110] S. Kang , A. Mansurov , T. Kurtanich , et al., “Engineered IL‐7 Synergizes With IL‐12 Immunotherapy to Prevent T Cell Exhaustion and Promote Memory Without Exacerbating Toxicity,” Science Advances 9 (2023): eadh9879, 10.1126/sciadv.adh9879. 38019919 PMC10686557

[111] Q. Zhao , J. Hu , L. Kong , et al., “FGL2‐Targeting T Cells Exhibit Antitumor Effects on Glioblastoma and Recruit Tumor‐Specific Brain‐Resident Memory T Cells,” Nature Communications 14 (2023): 735, 10.1038/s41467-023-36430-2. PMC9911733 36759517

[112] A. J. Ozga , M. T. Chow , and A. D. Luster , “Chemokines and the Immune Response to Cancer,” Immunity 54 (2021): 859–874, 10.1016/j.immuni.2021.01.012. 33838745 PMC8434759

[113] A. M. Vargason , A. C. Anselmo , and S. Mitragotri , “The Evolution of Commercial Drug Delivery Technologies,” Nature Biomedical Engineering 5 (2021): 951–967, 10.1038/s41551-021-00698-w. 33795852

[114] X. Peng , J. Fang , C. Lou , et al., “Engineered Nanoparticles for Precise Targeted Drug Delivery and Enhanced Therapeutic Efficacy in Cancer Immunotherapy,” Acta Pharmaceutica Sinica B 14 (2024): 3432–3456, 10.1016/j.apsb.2024.05.010. 39220871 PMC11365410

[115] Z. Xu , S. Liu , Y. Li , et al., “Engineering Strategies of Sequential Drug Delivery Systems for Combination Tumor Immunotherapy,” Acta Pharmaceutica Sinica B 15 (2025): 3951–3977, 10.1016/j.apsb.2025.05.039. 40893686 PMC12399215

[116] J. Galon and D. Bruni , “Approaches to Treat Immune Hot, Altered and Cold Tumours With Combination Immunotherapies,” Nature Reviews. Drug Discovery 18 (2019): 197–218, 10.1038/s41573-018-0007-y. 30610226

[117] B. Wu , B. Zhang , B. Li , H. Wu , and M. Jiang , “Cold and Hot Tumors: From Molecular Mechanisms to Targeted Therapy,” Signal Transduction and Targeted Therapy 9 (2024): 274, 10.1038/s41392-024-01979-x. 39420203 PMC11491057

[118] B. D. Shields , B. Koss , E. M. Taylor , et al., “Loss of E‐Cadherin Inhibits CD103 Antitumor Activity and Reduces Checkpoint Blockade Responsiveness in Melanoma,” Cancer Research 79 (2019): 1113–1123, 10.1158/0008-5472.CAN-18-1722. 30674537 PMC6420873

[119] S. Corgnac , I. Damei , G. Gros , et al., “Cancer Stem‐Like Cells Evade CD8(+)CD103(+) Tumor‐Resident Memory T (T(RM)) Lymphocytes by Initiating an Epithelial‐To‐Mesenchymal Transition Program in a Human Lung Tumor Model,” Journal for Immunotherapy of Cancer 10 (2022): e004527, 10.1136/jitc-2022-004527. 35418483 PMC9014106

[120] D. V. F. Tauriello , E. Sancho , and E. Batlle , “Overcoming TGFbeta‐Mediated Immune Evasion in Cancer,” Nature Reviews. Cancer 22 (2022): 25–44, 10.1038/s41568-021-00413-6. 34671117

[121] H. Kim , S. B. Lee , J. K. Myung , et al., “SLUG Is a Key Regulator of Epithelial‐Mesenchymal Transition in Pleomorphic Adenoma,” Laboratory Investigation 102 (2022): 631–640, 10.1038/s41374-022-00739-1. 35145202

[122] J. J. Park , M. H. Park , E. H. Oh , et al., “The p21‐Activated Kinase 4‐Slug Transcription Factor Axis Promotes Epithelial‐Mesenchymal Transition and Worsens Prognosis in Prostate Cancer,” Oncogene 37 (2018): 5147–5159, 10.1038/s41388-018-0327-8. 29849120

[123] P. S. Chowdhury , K. Chamoto , A. Kumar , and T. Honjo , “PPAR‐Induced Fatty Acid Oxidation in T Cells Increases the Number of Tumor‐Reactive CD8(+) T Cells and Facilitates Anti‐PD‐1 Therapy,” Cancer Immunology Research 6 (2018): 1375–1387, 10.1158/2326-6066.CIR-18-0095. 30143538

[124] Y. Simoni , E. Becht , M. Fehlings , et al., “Bystander CD8(+) T Cells Are Abundant and Phenotypically Distinct in Human Tumour Infiltrates,” Nature 557 (2018): 575–579, 10.1038/s41586-018-0130-2. 29769722

[125] J. Ning , N. V. Gavil , S. Wu , et al., “Functional Virus‐Specific Memory T Cells Survey Glioblastoma,” Cancer Immunology, Immunotherapy 71 (2022): 1863–1875, 10.1007/s00262-021-03125-w. 35001153 PMC9271132

[126] G. Oliveira , K. Stromhaug , S. Klaeger , et al., “Phenotype, Specificity and Avidity of Antitumour CD8(+) T Cells in Melanoma,” Nature 596 (2021): 119–125, 10.1038/s41586-021-03704-y. 34290406 PMC9187974

[127] P. C. Rosato , S. Wijeyesinghe , J. M. Stolley , et al., “Virus‐Specific Memory T Cells Populate Tumors and Can Be Repurposed for Tumor Immunotherapy,” Nature Communications 10 (2019): 567, 10.1038/s41467-019-08534-1. PMC6362136 30718505

[128] N. Cuburu , L. Bialkowski , S. M. Pontejo , et al., “Harnessing Anti‐Cytomegalovirus Immunity for Local Immunotherapy Against Solid Tumors,” Proceedings of the National Academy of Sciences of the United States of America 119 (2022): e2116738119, 10.1073/pnas.2116738119. 35749366 PMC9245622

[129] N. P. Restifo , M. E. Dudley , and S. A. Rosenberg , “Adoptive Immunotherapy for Cancer: Harnessing the T Cell Response,” Nature Reviews. Immunology 12 (2012): 269–281, 10.1038/nri3191. PMC6292222 22437939

[130] D. E. Ramirez , A. Mohamed , Y. H. Huang , and M. J. Turk , “In the Right Place at the Right Time: Tissue‐Resident Memory T Cells in Immunity to Cancer,” Current Opinion in Immunology 83 (2023): 102338, 10.1016/j.coi.2023.102338. 37229984 PMC10631801

[131] C. E. Weeden , V. Gayevskiy , C. Marceaux , et al., “Early Immune Pressure Initiated by Tissue‐Resident Memory T Cells Sculpts Tumor Evolution in Non‐Small Cell Lung Cancer,” Cancer Cell 41 (2023): 837–852.e6, 10.1016/j.ccell.2023.03.019. 37086716

